# Genome-wide predictors of NF-κB recruitment and transcriptional activity

**DOI:** 10.1186/s13040-015-0071-3

**Published:** 2015-11-26

**Authors:** Marcin Cieślik, Stefan Bekiranov

**Affiliations:** 1 Department of Biochemistry and Molecular Genetics, University of Virginia School of Medicine, Charlottesville, Virginia USA; 2 Michigan Center for Translational Pathology, University of Michigan Medical School, Ann Arbor, Michigan 48109 USA

## Abstract

**Background:**

Inducible transcription factors (TFs) mediate transcriptional responses to environmental cues. In response to multiple inflammatory signals active NF-κB dimers enter the nucleus and trigger cell-type-, and stimulus-specific transcriptional programs. Although much is known about NF-κB inducing pathways and about locus-specific mechanisms of transcriptional control, it is poorly understood how the pre-existing chromatin landscape determines NF-κB target selection and activation. Specifically, it is not known which epigenetic marks and pre-bound TFs serve genome-wide as positive (negative) cues for active NF-κB.

**Results:**

We applied multivariate and combinatorial data mining techniques on a comprehensive dataset of DNA methylation, DNase I hypersensitivity, eight epigenetic marks, and 34 TFs to arrive at genome-wide patterns that predict NF-κB binding. Strikingly, we observed NF-κB recruitment to accessible and nucleosome-bound sites. Within nucleosomal DNA NF-κB binding was primed by H3K4me1 and H2A.Z, but also hyper-methylated DNA outside of promoters and CpG-islands. Many of these predictors showed combinatorial cooperativity and statistically significant interactions. Recruitment to pre-accessible sites was more frequent and influenced by chromatin-associated TFs. We observed that specific TF-combinations are greatly enriched for (or depleted of) NF-κB binding events.

**Conclusions:**

We provide evidence of NF-κB binding within genomic regions that lack classical marks of activity. These pioneer binding events are relatively often associated with transcriptional regulation. Further, our predictive models indicate that specific combinations of epigenetic marks and transcription factors predetermine the NF-κB cistrome, supporting the feasibility of using statistical approaches to identify “histone codes”.

**Electronic supplementary material:**

The online version of this article (doi:10.1186/s13040-015-0071-3) contains supplementary material, which is available to authorized users.

## Background

Complex mechanisms have evolved to control gene expression in response to stimuli [1]. Signaling pathways bridge environmental signals with changes in gene expression patterns. The terminal transcription factors (TFs) of these pathways bind to specific regulatory regions and modulate gene expression through interactions with co-activators and co-repressors [2]. TFs need to locate their target sites within more than 3 billion base pairs of the human genome. The specificity of motif binding, accessibility of recognition sites, and expression of cell-type restricted cooperative binding partners, is believed to determine the fidelity of the transcriptional response (e.g. [3–5]). The canonical role of some TFs is to mediate responses to environmental cues (e.g. [6–8]), which converges on a relatively small number of TFs including CREB1 and the AP-1 and NF-κB families [9, 10]. The goal of this study was to computationally dissect the biological signals that pre-determine recruitment and transcriptional activation by NF-κB using logistic regression, non-negative matrix factorization, combinatorial enrichment analysis and statistical enrichment analysis.

The NF-κB-mediated response depends on the translocation of NF-κB proteins to the nucleus [11]. Selectivity of the transcriptional program is in part due to the specificity of different NF-κB homo- and hetero-dimers to distinct subsets of targets and cognate sequences [12–14], but also depends on the availability of tissue-restricted DNase I hypersensitive (DHS) sites [15]. These differences are among the primary determinants of cell-type identity, but can change in response to the environment and in pathologies [16–18].

Mechanisms of chromatin-mediated TF recruitment are well understood for the ligand-induced nuclear glucocorticoid receptor (GR) [19]. GR target-genes are often induced (or repressed) rapidly in response to hormone treatment [20]. It has been shown that GR binding at target promoters is almost exclusively associated with chromatin that is hypersensitive prior to hormone treatment [21]. These regions of accessible chromatin have been shown to be maintained by AP-1, which was required for GR-mediated response [22, 23].

In stark contrast to GR, “pioneer” transcription factors, such as PU.1 or *FOXA1*, are typically lineage-restricted and transcriptionally regulated [24]. They have the ability to bind nucleosomal DNA and to reduce nucleosome occupancy [25–27]. The functional consequence of this is the transcriptional activation of enhancers and promoters [28]. *FOXA1* has been shown to be recruited to locally hypo-methylated sites within broader hyper-methylated regions [29]. These unmethylated CpG windows have been reported to complement bivalent domains in defining tissue-specific enhancers [30]. Importantly, it was recently shown that the pluripotency TFs *OCT4*, *SOX2*, and *KLF4*, have chromatin-binding characteristics very similar to classic pioneers [31, 32]. These studies highlighted that pioneer TFs have a similar role in chromatin remodeling, during development, cellular reprogramming, and transcriptional activation [33–37].

Transcriptional activation by NF-κB and its recruitment to chromatin appears to involve multiple mechanisms and results in a complex chromatin binding pattern and multiphasic transcriptional kinetics [38, 39]. According to the current model, differences in NF-κB binding are largely determined by the chromatin state [40], but specific targeting signals are unknown. Some target sites are bound immediately, whereas binding to others is delayed and ostensibly depends on prior chromatin remodeling [41–43]. It has been proposed that immediate binding sites are constitutive i.e. associated with genes induced in response to a broad range of stimuli. On the other hand, delayed binding is thought to be regulated through the cell type specific immediate response and induction of pathways that result in the remodeling of chromatin [9, 40, 44].

TNF-induced p65 (RELA or NFKB3, an NF-κB subunit) binding has been mapped genome-wide in a number of cells lines (THP-1, HeLa, A549, dendritic cells [15, 45, 46]). These data provided evidence that p65 is recruited to tens of thousands of cell-type specific loci [35, 47, 48]. Strikingly, a substantial number (~17 %) of p65 binding events occurred outside of pre-accessible chromatin [15, 49–52].

Despite these advances, much work remains to be done in order to better understand how inducible TFs including p65 are recruited to their eventual binding sites [40]. Here, we leveraged statistical machine learning on large integrated functional genomic data sets generated by ENCODE and other groups in A549 cells (a non-small cell lung cancer cell line and model for the study of inflammatory signaling in cancer) to assess the extent to which the majority of observed binding events are opportunistic or guided by specific signals [53–56]. Specifically, we analyzed binding sites for 34 TFs and two control tracks, digital genomic foot-printing (DGF) [57], DNA methylation (RRBS) [58], ChIP-seq [59] for 8 epigenetic marks, and RNA-seq [60] data. To arrive at predictive rules that determine which sites are likely to recruit p65, we statistically evaluated the individual and cooperative impact of NF-κB motifs, DNA methylation, DNase I hypersensitivity, histone modifications, and pre-bound TFs on p65 binding. However, genome-wide patterns of epigenetic features are noisy, complex, and highly correlated. Different approaches are required for chromatin marks and TFs due to their continuous (quantitative signals) and discrete (sites along the genome) nature, respectively. We found that TF binding was sufficiently well captured through binary variables (i.e., site present or absent), whereas epigenetic marks require quantitative features. To discover rules that govern the p65 cistrome (i.e., factors and states of the chromatin that recruit p65) diverse computational and statistical techniques had to be applied and developed including logistic regression based approaches—presence or absence of p65 binding as a function of quantitative features including histone modification levels. However, the identification of factors that cooperate to recruit p65 using regression-based approaches is particularly challenging due to the co-linearity of many of the input variables, leading to potentially meaningless regression coefficients. Consequently, we developed and applied a matrix factorization approach and applied it to histone modification data, which identifies “codes” of co-occurring marks which tend to be less correlated and yield more meaningful, interpretable regression coefficients. Moreover, while we analyzed the ability of TFs to recruit p65 one at a time (univariate statistical enrichment analysis), TFs are known to co-recruit other factors and likely work in a cooperative, combinatorial fashion in further recruitment of factors like p65 upon TNF stimulation. Consequently, we developed and applied combinatorial enrichment/association methods that uncovered combinations of TFs that recruit p65. Predictive clusters of TFs and synergistic patterns of histone marks, were indeed captured through “greedy” association rules and matrix factorization, respectively, indicating the existence of a strong combinatorial epigenetic code. The codes were often context dependent, which suggests that datasets with higher temporal and spatial resolution will enable their further refinement. Our computational approach reveals that p65 binding occurs within accessible and inaccessible chromatin regions, hence suggesting that p65 may function as a pioneer TF. Within accessible regions p65 recruitment is significantly predicted by a number of chromatin features and their combinatorial interactions, revealing an intricate yet specific set of p65-targeting epigenetic codes.

## Methods

### Peak calling

TF peaks were called using PeakRanger [61] version 1.16, which has been shown to yield above average spatial accuracy compared to other peak callers, using the A549 ChIP-seq control track provided by ENCODE. Due to the large number of processed data sets we used the tool consistently with all default parameters. DNase I hypersensitive (DHS) [62] sites from DGF sequencing were also called using PeakRanger and the suitability of this method was visually inspected.

### Motif analysis

*De novo* motif discovery was done within p65 peaks (see Peak Calling) that did not overlap another TF peak or DHS site using MEME-chip with all default settings [63]. The recovered NF-κB motif, which closely matches the canonical sequence, was used to scan all genomic sites using tomtom [64] (see Regulatory sites definition).

### Site conservation

We probed evolutionary conservation of regulatory sites *via* PhastCons [65] conservation scores for placental mammals, which limited our focus to conservation across mammalian vertebrates and were obtained from the UCSC genome browser track: phastCons46way.placental.

### Known NF-κB targets

Refseq ids of known targets were retrieved from: http://www.bu.edu/nf-kb/gene-resources/target-genes/. Accessed 23 Nov 2015.

### CpG islands

To classify genomic sites into CpG-islands (CGI) and non-CGI, we used two complimentary CGI definitions. The first set of CGIs was obtained from http://rafalab.jhsph.edu/CGI/-model-based-cpg-islands-hg19.txt[66]. The second set was from the UCSC genome browser cpgIslandExt.gz [67]. We defined a site as CGI or non-CGI if both CGI definitions were in agreement.

### DNA methylation

Experimental methylation levels at CpG sites were provided by ENCODE. The data was generated using the reduced representation bisulfite sequencing technique (RRBS) (HAIB Methyl RRBS Track Protocol). Two replicates were available probing methylation at over 1.5 M sites. These sites are biased towards MspI cleavage sites, and high GC content, but cover the majority of promoters and CGIs. We kept only sites that were present in both replicates and had ten reads coverage. We classified them depending on methylation range (0 % < 25 % < 75 % < 100 %) as “low”, “medium”, “high” [68].

### Regulatory sites definition

For statistical analyses and significance tests we exclude parts of the genome that appear to have no regulatory activity in either TNF-stimulated or unstimualted A549 cells. This allows us to evaluate statistical enrichments more stringently since we are omitting a large portion of the genome that had low *a priori* likelihood of being bound by p65. We base our definition of “regulatory sites” conservatively on TF-binding and DNase I hypersensitivity in unstimulated cells. We merge overlapping peaks of TFs into sites. Consequently, each site contains one or more TF peaks which overlap or are directly adjacent (no gap). Specifically, we use the cluster tree implementation from bx.python (https://bitbucket.org/james_taylor/bx-python/) to find the set of all “regulatory sites”.

### Regulatory sites classification

We defined several sub-types (classes) of regulatory sites. First, we identified “inaccessible sites” as occupied by p65 in TNF-stimulated cells but not other TFs or DNase I hypersensitive sites in unstimulated cells. All remaining, regulatory sites were termed “accessible” as they were either occupied by a TF in unstimulated cells or DNase I hypersensitive. First, regulatory sites were classified as TSS-proximal or TSS-distal. If a site overlapped any TSS known to GENCODE v14 it was defined as TSS-proximal. Next we sub-classified proximal or distal sites based on number of TFs bound (occupancy): stage 1 TSS-proximal bound 1–2 TFs; stage 1 TSS-distal - bound by exactly one TF; stage 2 TSS-distal - bound by 2 to 4 TFs. The following marks: CTCF, DNase I hypersensitivity, and RAD21 (cohesin) did not contribute to the occupancy count.

### Enrichment profiles

Positional enrichment profiles for epigenetic marks and PhastCons scores were generated using a modified version of the ACT.py using the tool’s default setting at 20 bp resolution [69].

### ChIP-seq data scaling

Within regulatory sites reads counts have been obtained using: genomic_overlaps count -i, or bedtools intersect -c from the aligned ChIP-seq datasets. These raw read-counts were normalized by region length. And scaled to the 0 to 1 range using a sigmoid function, such that the scaling is approximately linear up to the 95th percentile [70].

### Logistic regression

All models predicted binary p65 occupancy from levels of scaled epigenetic marks, epigenetic combinatorial patterns (see Combinatorial NMF “codes”), and binary p65 motif occurrence. The individual models differed in the number of included independent variables as described in the text. All models were trained according to the same scheme using scikits-learn [71]. 20 % of the observations were withheld as a test set. On the 80 % training set we used 10-fold cross-validation to select the best prior regularization parameters (*L*_*1*_ or *L*_*2*_ and *C*) using the Matthews correlation coefficient (MCC) as a balanced performance measure [72], and the prediction cut-off *p*_*c*_ *= 0.5*. The best cross-validated model was further tuned by scanning for a p_c_ that maximized the model’s MCC on the training set. The tuned model was benchmarked against the test set; we report both area under curve (AUC) and MCC.

### Epigenetic mark correlations

Correlations between scaled epigenetic marks were calculated as Spearman’s rank correlation coefficient.

### Epigenetic mark interactions

We have chosen the geometric mean (GM) to represent the joint magnitude of two marks. It is the square root transformation of the product of two magnitudes and scales the data to resemble a normal distribution.

### Association between continuous levels and binary response

To assess the strength of association between epigenetic marks (or mark interactions) with p65 binding we calculated enrichment *p*-values *via* the random set (RS) statistical test [73]. The RS test statistic has a large magnitude, and corresponding small *p*-value, only if sites occupied by p65 have higher levels of marks, codes, expression, *etc.* than sites selected at random.

### Combinatorial NMF “codes” of epigenetic marks

We used the NMF algorithm [74, 75] to decompose “chromatin patterns” at sub-classes of regulatory sites (see Regulatory sites classification) into small combinatorial “codes” of epigenetic marks [76]. Intuitively, codes capture correlations between histone modifications that are either global or local (valid for a subset of sites). Within each code values of individual histone modifications are tied, which is a proxy for their average relative abundance. Each regulatory site is associated with a weight for each code, the complete “chromatin pattern” (see ChIP-seq data scaling) is reconstructed by summing over codes multiplied by their weights. We validate that regression on code weights has desirable properties over regression on all scaled levels of all individual marks (see Additional file 1: Methods).

### Combinatorial chromatin clusters of transcription factors

We used two approaches to discover chromatin-based combinatorial clusters of transcription factors; “TF-clusters” that are associated with p65 recruitment. This is a combinatorial optimization problem as the number of possible combinations of 30+ TFs is large. The first approach (TGC) directly relied on the significance of the statistical enrichment, Fisher’s exact test (FET), and greedily “expanded” TF-clusters if they improved the p-value (association strength) by 0.2 (log2-odds), but were also relatively frequently observed genome-wide (at least 40 instances). The second approach, called “top-k non-redundant association rule” (TNR, [77]) was less biased in that it found the most “important” non-redundant associations between combinations of TFs, from which we selected those that predicted p65 binding (see Additional file 1: Methods).

### Linking genes, TSS-proximal and TSS-distal sites

To assign TSS-distal to TSS-proximal regulatory sites we followed a probabilistic approach that involves both distance restraints (100 kb) and the similarity of TF binding patterns (based on [37]). To assign regulatory sites to protein-coding genes we use a simple overlap criterion for TSS-proximal sites, and a 100 kb distance limit for enhancers (see Additional file 1: Methods).

### Differential expression

We used the DESeq [78] algorithm to identify differentially expressed (up- or down-regulated) GENCODE genes. We obtained raw exon-based expression estimates using the htseq-count defaults (see Additional file 1: Methods).

### Statistical analysis of proportions

Ninety five percent confidence intervals for the proportion of upregulated genes and statistical significance of the difference between two proportions have been estimated using the methods suggested by [79] as implemented in the R-package binom.

## Results

### p65 is recruited to “latent” and “primed” enhancers

**Fig. 1:**
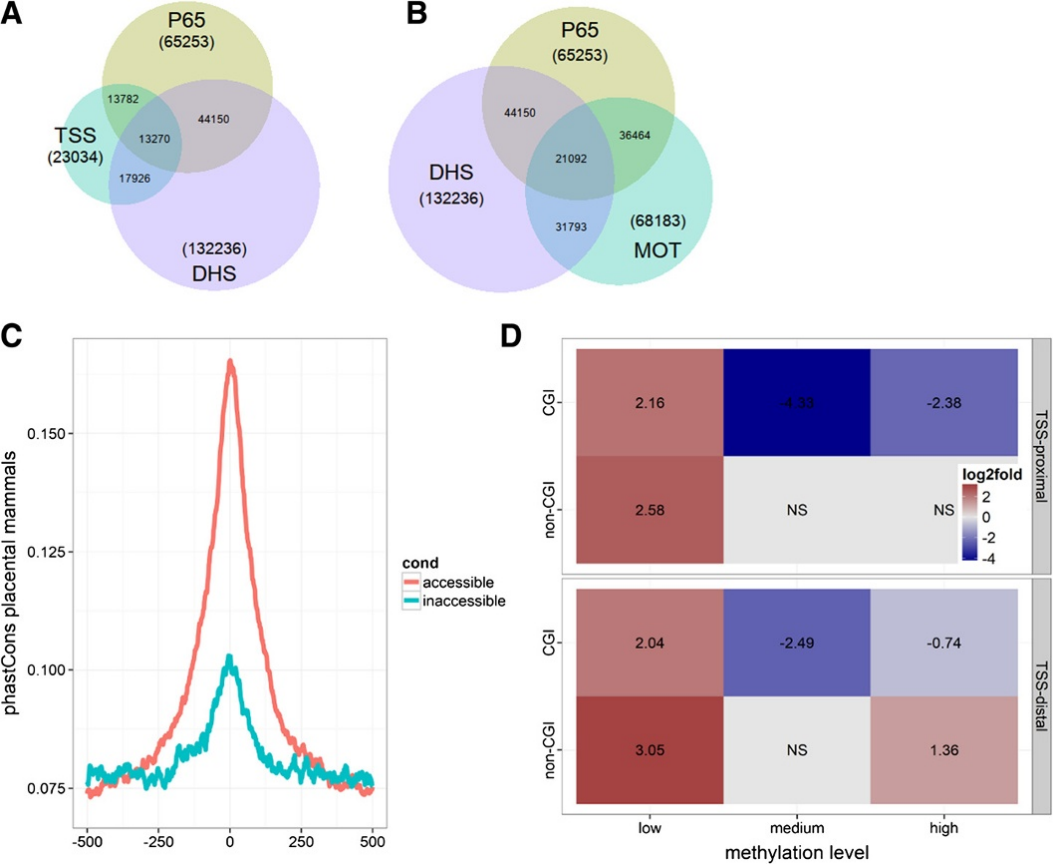
General characterization of genome-wide p65 binding. **a** Recruitment of p65 relative to transcription start sites (TSS) and DNase I hypersensitive (DHS) sites. Numbers in parentheses are totals of sites of a given class. **b** Likewise, recruitment of p65 relative to DHS and NF-κB motif (MOT) occurrence. **c** PhastCons placental mammals conservation scores at p65 summits within accessible and inaccessible regulatory sites (see Methods). **d** Role of CpG methylation in p65 binding. Significant enrichment (red) or depletion (blue) of p65 binding depending on localization (TSS-proximal, TSS-distal), CpG-island (CGI) status (CGI, non-CGI), methylation level (low, medium, high) (see Methods). All shown log-odds are significant (FET *p*-value < 1e-4)

According to the current model, p65 (RELA) is rapidly recruited to mostly constitutively accessible sites [15, 40]. To assess this on a genome-wide scale we determined the overlap between DNase I hypersensitive (DHS) sites and post-induction p65 peaks (Fig. 1a–b). DHS regions are reliable indicators of DNA binding by the majority of TFs [57, 80–82]. We find a total of 70,146 p65 peaks at a 1 % FDR, which reduce to 65,253 after collapsing into larger regulatory sites (Methods). We have chosen to include all peaks which cannot be explained by experimental noise [53, 83]. Notably, of the approximately 400 known, well-annotated NF-κB target genes (see Methods) we find 150 contain a peak in their promoter-proximal region (see Additional file 2: Data). We find a surprisingly large number (21,103) of p65 binding events outside of pre-existing DHS sites, mostly outside (20,591) of promoter-proximal regions (Fig. 1a; Fig. 1a and b are similar to Venn diagrams and show the total overlaps between p65, promoters, DHS and NF-κB motifs). In total, 32 % off all p65 peaks are outside DHS sites, while 33 % of all (132,236) DHS sites show enrichment of p65. To rule-out the possibility that the majority of p65 binding is non-specific and opportunistic due to saturation [53, 83, 84], we assessed the overlap of experimental peaks with occurrences of the NF-κB motif (Fig. 1b). Overall, of the 65,253 p65-positive regions 36,464 (56 %) have an NF-κB motif (Methods). Strikingly, this proportion was significantly higher for p65 peaks outside DHS sites 73 %, compared to 48 % in nucleosome-free regions. Next, we attempted to identify other proteins that are co-recruited with p65 to the nucleosome-bound sites by applying *de novo* motif discovery. However, the only detectable motif was the canonical NF-κB sequence (Additional file 1: Figure S1A). To test whether p65 binding outside of nucleosome-depleted regions is likely “functional” we probed for evolutionary conservation (Methods). We found evidence for purifying selection at p65 summits within and outside DHS sites (Fig. 1c). We observed significantly stronger conservation at summits within constitutive DHS sites. Which is expected since lineage-specific regulatory sequences are not well conserved across mammals [85].

#### Recruitment to hyper-methylated distal regulatory sites (statistical enrichment analysis)

Next, since TFs differ markedly in their ability to bind methylated DNA, we asked whether CpG-methylation influences p65 recruitment propensities. We assessed the overlap between p65 peaks and CpG methylation status at promoter and putative enhancer regions divided into CpG-islands (CGIs) and non-CpG-islands (non-CGIs) (Fig. 1d). In normal cells most CGIs are constitutively hypo-methylated. Conversely, non-CGIs, which include gene body and enhancer CpGs, have dynamic methylation levels [86]. Cancer cells in general are characterized by global losses of non-CGI methylation and focal increases in CGI methylation [87]. Importantly, LPS primary response genes tend to have CpG-rich constitutively accessible promoters [44]. We found that p65 recruitment is sensitive to methylation status both at promoter-proximal and distal regulatory regions and depends on the CGI context. As expected, hypo-methylated CGI sites showed an increased propensity for p65 binding, whereas methylated CGIs were depleted of p65. The effect was more pronounced at TSS-proximal sites. Hypo-methylated non-CGI promoters were likewise enriched for p65, however we also observed a modest but significant (2.5-fold, (Fisher’s Exact Test) FET *p*-value 8.38e-73) enrichment (over that expected by chance) for p65 binding at hyper-methylated, non-CpG, TSS-distal regions. This is unexpected given that, genome-wide, p65 preferentially binds to promoters (11-fold enrichment), CpG-islands, and hypo-methylated sites (16-fold). Why would DNA methylation at a class of non-CpG, promoter-distal regions increase p65 binding propensity? In stark contrast to CGIs, at CpG-poor sites hyper-methylation is correlated with DNase I hypersensitivity (2-fold, p 7.28e-64). However, even after controlling for DHS status hyper-methylated regions are still enriched for p65 peaks in DHS-positive (1.65-fold, p 5.91e-13) and DHS-negative (3-fold, p 2.25e-42) subsets of non-CpG, promoter-distal sites.

#### Rapid recruitment to inaccessible heterochromatic regions

**Fig. 2:**
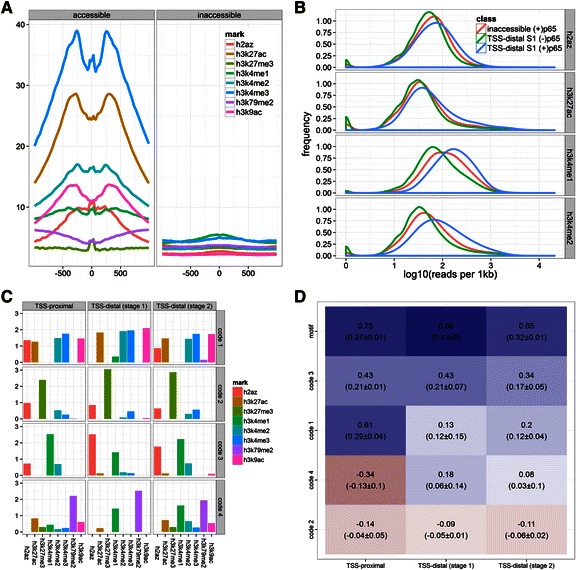
Distribution of epigenetic marks at p65 summits and p65 recruitment by combinations of frequently co-occurring epigenetic marks. **a** Enrichment profiles of all 8 epigenetic marks around p65 peak summits at accessible and inaccessible sites. Enrichment levels should be compared for a single mark between the two classes of sites, but not between different marks for a single class, as magnitudes of ChIP-seq peaks are, in general, not comparable. **b** Densities of epigenetic marks at p65 peaks within inaccessible regions compared to accessible *stage 1* (S2) TSS-distal regulatory sites, bound by p65 (+) or not (−) (see Methods). We applied non-negative matrix factorization (NMF) to matrices of scaled levels of epigenetic marks separately for the three classes of regulatory sites (TSS-proximal, TSS-distal *stage 1*, TSS-distal *stage 2*). For each class the method returned four combinations (codes) of strongly associated epigenetic marks. Next, we trained logistic regression models to predict p65 binding from locus-specific weights for each code and NF-κB motif occurrence (mot). **c** Within each code, the loadings of marks are tied, which reflects their most frequent relative abundances, and is also a measure of relative mark importance. Equivalent codes between classes of sites show differences in mark loadings. **d** Heatmap of standardized regression slopes for the models’ parameters, which include the four codes and motif presence (mot). Mean and standard deviation of slopes obtained from dropping each of the other covariates from the model is indicated in brackets, and is a measure of the robustness of the estimate to model specification (see Methods and Additional file 1: Methods)

We hypothesized that p65 recruitment to both DHS-positive and DHS-negative regions is guided, to some extent, by epigenetic marks [26, 88]. We used DHS sites and TF peaks to identify all genomic “regulatory sites” in TNF-stimulated and unstimulated A549 cells (Methods) and classified them as either “accessible” or “inaccessible”. The accessible sites were either, strictly DNase I hypersensitive, or enriched for any of the 34 TFs for which data was available (Methods). Surprisingly, we found that 16,065 p65 summits were within inaccessible regions. One concern is that these sites are possibly non-functional. We again found evidence for sequence conservation at p65 summits within inaccessible sites (Fig. 1c). We also find a large fraction of the 16,065 sites contain one or more NF-κB motifs (Fig. 1b). As we discuss below, we find p65-dependent transcriptional induction displays a strong monotonic downward trend as a function of the number of TFs bound at promoters (see Fig. 5). Promoters with no TFs bound showed the strongest induction consistent with this trend. Importantly, many of these promoters do not contain a DHS, further arguing that these inaccessible sites are functional. Finally, we compared chromatin enrichment profiles between the two classes of sites and found that accessible sites were enriched for epigenetic marks typically associated with permissive or active chromatin, while inaccessible sites showed no significant enrichment (Fig. 2a).

#### Differences in histone modification levels at distinct classes of p65 binding sites (comparison of distributions)

To investigate this in more detail we compared histone modification levels between p65-bound and unbound sites. For this and all subsequent analyses we focus on three classes of accessible regulatory sites. (1) TSS-proximal sites with at most 2 TFs pre-bound; (2) DHS-negative TSS-distal sites with at most 1 TF pre-bound; and (3) DHS-positive TSS-distal sites with 2 to 4 TFs pre-bound. We will refer to these classes as *stage 1* promoters, *stage 1* enhancers, and *stage 2* enhancers, respectively. The rationale behind this classification is to separate the earliest detectable high-confidence regulatory regions (single TF), from enhancers that recruited co-factors (up to 4 TFs) and from fully assembled enhancers.

We found that sites that recruit p65 have higher levels of several histone modifications, H3K4me1 in particular (*stage 1* Fig. 2b, *stage* 2 Additional file 1: Figure S2). However, the differences were pronounced only at stage 2 enhancers, which are almost exclusively accessible sites (Additional file 1: Figure S2). Within inaccessible regions p65 site selection was weakly correlated with H3K4me1 and H3K4me2, but almost fully independent of H3K27ac (Fig. 2b). At accessible sites enrichment profiles for several marks have a bimodal shape around the peak summit, which is the most likely location of the NF-κB motif (Additional file 1: Figure S1B) [47, 89]. On the other hand, H2A.Z, a histone variant associated with regulatory regions [90], has a single peak over the p65 summit. This suggests that the H2A.Z-containing nucleosome is centered on the NF-κB element.

#### Pioneering recruitment of p65 to latent and epigenetically silent enhancers

Together, these results suggest that within 30 min of induction (i.e., 30 min after initial TNF treatment; see [46] for experimental details), p65 is being recruited to both, DNase I hypersensitive and nucleosome-bound sites. Although the recruitment to DHS sites is qualitatively stronger (Additional file 1: Figure S3) and occurs more frequently (7.1-fold, p 0), p65 binding to nucleosome-bound DNA appears to be more specific as judged by the occurrence of the NF-κB motif. Constitutively accessible sites typically harbor clusters of multiple TF recognition elements [53, 81], which might suggest that the higher conservation of accessible sites is due to purifying selection on motifs of other TFs. Most strikingly, outside of promoters and CpG islands, p65 binds preferentially to methylated sites; which brings to mind the methyl-DNA binding capabilities of FOXA1, a “pioneer” transcription factor [29, 91, 92]. In summary, we found that p65 binding within inaccessible regions occurred both at epigenetically silent “latent” (i.e., H3K4me1 low) and epigenetically “primed” enhancers (i.e., H3K4me1 high, and/or DNase I hyper-sensitive) [48, 93].

### Epigenetic marks are quantitative predictors of p65 recruitment

When transcription factor binding and histone modification levels are mapped in the same state (cell line, condition) they appear highly correlated. However, whether epigenetic marks are truly useful predictors of TF-binding is disputed (e.g. [94–96]). In particular it is not well established, whether histone modifications confer binding specificity, and if TFs prefer sites with a specific chromatin pattern [88]. We decided to test this hypothesis by using histone modification levels prior to stimulation to predict which sites will recruit p65. Since there is no consensus regarding how to define enhancers from epigenetic marks (e.g. [89]) we favored a conservative approach and identified cis-regulatory regions based on the presence of at least one TF and/or DNase susceptibility. Only a small fraction of all sites (~5 %, 10,625) will recruit p65 on stimulation.

#### Univariate predictive modeling of p65 recruitment (logistic regression analysis)

We tested whether p65 binding can be predicted from histone modification levels, either on their own, or together with occurrences of the κB recognition element (Table 1). We modeled the probability of p65 recruitment using a logistic function using histone modifications and motif occurrences as predictors (see Methods). We found that motif-based prediction was universally improved by the inclusion of any histone modification, with a substantial average gain of Δ0.055 area under ROC (AUC). The improvement was highest at promoters, on average by Δ0.072 AUC with six of the eight histone modifications reaching Δ0.10 AUC. It is remarkable that at TSS-proximal sites H2A.Z and H3K4me2 are better predictors of p65 recruitment than the occurrence of the p65 motif – 0.72 *vs* 0.69 AUC, respectively. The binding of p65 at *stage 1* enhancers sites is most strongly determined by the presence of the NF-κB motif (0.79 AUC). This score is most significantly improved by H3K4me1 to 0.86 AUC, which along with H3K4me2 and the p65 motif yield the best-performing models overall. At enhancers with several cofactors, neither motif-occurrence, nor histone modifications were individually sufficient to model p65 binding (max 0.63 AUC) and even the best-performing combined model barely exceeded 0.70 AUC. The predictive performance of histone acetylations was low but similar at *stage 1* and *stage 2* enhancers. Conversely, lysine methylations, which significantly determined p65 binding at *stage 1* enhancers, were less relevant at *stage 2* enhancers (Table 1).

**Table 1:** Performance of epigenetic marks in the prediction of p65 binding

	Prox	Dist S1	Dist S2	mean	∆mot
h2az	0.72	0.61	0.60	0.64	
h2az + mot	0.80	0.83	0.69	0.77	0.13
h3k27ac	0.70	0.64	0.61	0.65	
h3k27ac + mot	0.80	0.84	0.69	0.78	0.13
h3k27me3	0.52	0.53	0.54	0.53	
h3k27me3 + mot	0.70	0.80	0.66	0.72	0.19
h3k4me1	0.59	0.70	0.59	0.63	
h3k4me1 + mot	0.74	0.86	0.68	0.76	0.14
h3k4me2	0.72	0.70	0.61	0.67	
h3k4me2 + mot	0.80	0.86	0.70	0.79	0.11
h3k4me3	0.70	0.64	0.58	0.64	
h3k4me3 + mot	0.80	0.84	0.68	0.77	0.13
h3k79me2	0.50	0.53	0.53	0.52	
h3k79me2 + mot	0.69	0.80	0.65	0.71	0.20
h3k9ac	0.69	0.64	0.63	0.65	
h3k9ac + mot	0.79	0.84	0.71	0.78	0.13
mot	0.69	0.79	0.63	0.71	

Predictive performance (AUC – area under curve) of single-mark logistic regression models, with optional inclusion of binary NF-κB motif presence (+mot), on the binding of p65 at three classes of sites: Prox – TSS-proximal; Dist S1 – TSS-distal (stage 1); Dist S2 – TSS-distal (stage 2); mean – arithmetic average performance for all classes; ∆mot – arithmetic average improvement of the (+mot) models

We have shown that even after controlling for DNase I hypersensitivity, TF occupancy, and motif occurrence, specific epigenetic marks determine p65 recruitment. It was previously shown that H3K4me1 is involved in the priming of enhancers for activation in multiple lineages [97]. H3K4me1 depositions are relatively stable [89], and their presence is thought to be determined by lineage-restricted “pioneer” transcription factors, such as PU.1 [29, 35, 47]. It has been postulated that this defines lineage-restricted enhancers and creates binding sites for additional inducible TFs [37, 98]. The finding that p65 recruitment to *stage 1* enhancers was correlated with H3K4me1 levels is consistent with the current interpretation of H3K4me1 as a mark of regulatory regions primed for activation [97, 99–101]. Histone acetylations are associated with active enhancers [100, 102], but are also highly correlated with DNase I hypersensitivity and other permissive histone marks [103]. In line with previous findings which determined an important role of H2A.Z in the regulation of transcription [104–106], our results suggest that H2A.Z-marked promoters are poised for increased TF binding (Additional file 1: Figure S4).

### Context-dependent roles of epigenetic marks in p65 recruitment

It has been argued that the co-occurrence of multiple epigenetic marks is necessary for specific chromatin-triggered processes [107, 108] and that combinations of multiple marks are required to reliably identify tissue-specific enhancers and direct TFs to specific chromatin regions [88]. We reasoned that p65 site selection could be further refined by cooperative action between multiple marks. To discover these synergistic effects we built a predictive model of synergistic p65 recruitment. This model was augmented by more standard correlative descriptions of genome-wide associations (Additional file 1: Figure S5).

#### Synergism and correlation between predictive chromatin features

Through correlative analyses (Additional file 1: Text) we found that canonical active marks (H3K9ac, H3K27ac, H3K4me3) were involved in synergistic interactions with H3K4me1 at promoters and H2A.Z at enhancers (Additional file 1: Figure S5). At promoters, high levels of H3K4me1 and active marks were mutually exclusive (Additional file 1: Figure S5A), and H3K4me1 was unable to recruit p65 on its own. This suggests that mono-methylation is only an intermediate in the further activation of promoters [109]. Conversely, at enhancers H3K4me1 has an independent role in p65 recruitment and appears to be required for H3K9ac enrichment (Additional file 1: Figure S5B). Although, p65 recruitment was weakly associated with H2A.Z [110] on its own (Table 1) we observed strong synergism between H2A.Z and other active marks at nucleosome-bound, but not nucleosome-free, enhancers (Additional file 1: Figure S5B).

#### Multivariate predictive modeling using non-negative matrix factorization (NMF)

To construct the predictive model we used non-negative matrix factorization (NMF) [111] to decompose “chromatin patterns” at TSS-proximal and TSS-distal regulatory regions into fundamental additive parts we refer to as “codes”. The method is explained in detail in the (Additional file 1: Methods) [76]. Briefly, we construct an “epigenetic matrix” *V*, which is factored *via* NMF into matrices *W* and *H. V* has rows which are regulatory sites and columns which are epigenetic marks. Elements of *V* are normalized levels of a specific mark at a certain site. When multiplied, matrices *W* and *H* result in an approximation of *V*. Matrix *H* contains a small number of rows, each row represents a combination of marks that frequently co-occur in subsets of genomic regions (codes). Matrix *W* contains weights on how to reconstruct each row of *V* using the codes in *H*.

We applied the algorithm separately on each of the three classes of regulatory sites and discovered four, largely equivalent, combinatorial patterns (Fig. 2c). The equivalent codes from different regions showed quantitative differences in mark values, which reflect differences in the relative levels and importance of marks within a pattern [76]. The first code subsumes the permissive epigenetic marks (H3K9ac, H3K27ac, H3K4me2, H3K4me3) (Additional file 1: Figure S6A). The remaining three codes capture context-dependent patterns, which is a particular strength of NMF [111, 112]. Globally H2A.Z and H3K4me1 are only weakly correlated (Additional file 1: Figure S6A), but they were hypothesized to co-occur at a subset of primed enhancers [101] and this exact pattern is captured through code 3. Another subclass of enhancers is silenced by the presence of the repressive H3K27me3 mark [99, 100]. Code 2 has positive values for H3K27me3 and H2A.Z, which captures their dependence at both promoters and enhancers. The last pattern is related to H3K79me2. At TSS-proximal regions this mark is associated with transcription elongation by RNAPII [113] and is relatively independent of other histone modifications (Additional file 1: Figure S6A). Conversely, within TSS-distal regulatory regions H3K79me2 is tied to the enhancer mark H3K4me1, which suggests that the code is largely specific to enhancers within introns of actively transcribed genes.

#### Signals of p65-targeting “histone codes” (logistic regression analysis using NMF “code” weights as predictors)

The unique “chromatin pattern” at each locus is reconstructed from prototypical codes and locus-specific weights. The codes are simply tied levels of specific mark combinations, the weights reflect the importance of a code at a locus. Together, this allows us to quantitatively link combinatorial epigenetic patterns to p65 recruitment using logistic regression (Methods). We modeled the probability of p65 binding as a function of the code weights and motif occurrence. The recruitment of p65 is most easily predicted at promoters (AUC = 0.82, MCC = 0.42) followed by *stage 1* (AUC = 0.85, MCC = 0.32), and *stage 2* (AUC = 0.71, MCC = 0.31) enhancers. We found that some codes were robust predictors of p65 binding (Fig. 2d, Additional file 1: Methods). Motif presence has the largest standardized coefficient (log-odds ratio, LOR). However, as few as 7.4 % of *stage 1* TSS-distal sites contain a p65 motif, which means that the large 0.86 LOR (2.4 fold) applies only to a rare subset of the population of sites [114]. We found that code 3 (Fig. 2c), was an important predictor at *stage 1* enhancers (Fig. 2d). Code 4 showed opposite roles in the recruitment of p65 to promoter and enhancer sites. This counter-intuitive observation is largely explained by differences in the code itself. At *stage 1* distal sites the code ties levels of H3K79me2 and H3K4me1, whereas at proximal sites it is driven by H3K79me2 alone. Still, it appears that promoters that have successfully recruited RNAPII and engaged in elongation – as judged by higher levels of H3K79me2 – are less likely to recruit the transcriptional activator p65. The “repressive” code 2 had only a weakly negative effect on p65 binding. This suggests that the repressive role of H3K27me3 can be mostly attributed to the inverse correlation with H3K27ac and other “active” marks (Additional file 1: Figure S6A), but not H3K27me3 itself (code 1).

Together, these results reveal context-dependent roles of histone modifications in p65 recruitment Further, we found that H2A.Z in the context of H3K4me1 (code 3) is the strongest epigenetic predictor of p65 recruitment to TSS-distal sites, whereas in the context of H3K27me3 (code 2) it is slightly impeding. H3K27me3 deposition has been shown to be dependent on H2A.Z [115], whereas the mechanism that lead to H3K4me1 and H2A.Z co-occurrence are poorly understood. Co-operativity of H2A.Z with other “active” marks (code 1) (Additional file 1: Figure S5B), agrees with the observation that codes 1 and 3 are relatively uncorrelated (Additional file 1: Figure S6B). The co-occurrence of H2A.Z and H3K4me1 at a relatively large subset of enhancers (code 2) further supports the emerging interpretation of the H2A.Z/H3K4me1 enhancer pattern as priming enhancers for activation [101].

### p65 site selection is guided by the presence of specific TFs and overall occupancy

While the predictive performance of the code-based models is considerably better than relying just on one mark and motif presence, the predictions are far from perfect (Table 1). The models’ sensitivity (i.e., percentage of correctly predicted p65 binding events) with respect to p65-bound sites is low (20 %) at *stage 1* enhancers, and modest at *stage 2* enhancers (34 %). This is still remarkable given that less than 2 % of *stage 1* sites are bound by p65. It has been widely accepted that tissue specific gene expression is achieved through combinatorial interactions among transcription factors [116, 117]. We reasoned that the presence or absence of chromatin-associated proteins, transcription factors, and their combinations could provide an independent set of cues for p65 site selection.

**Fig. 3:**
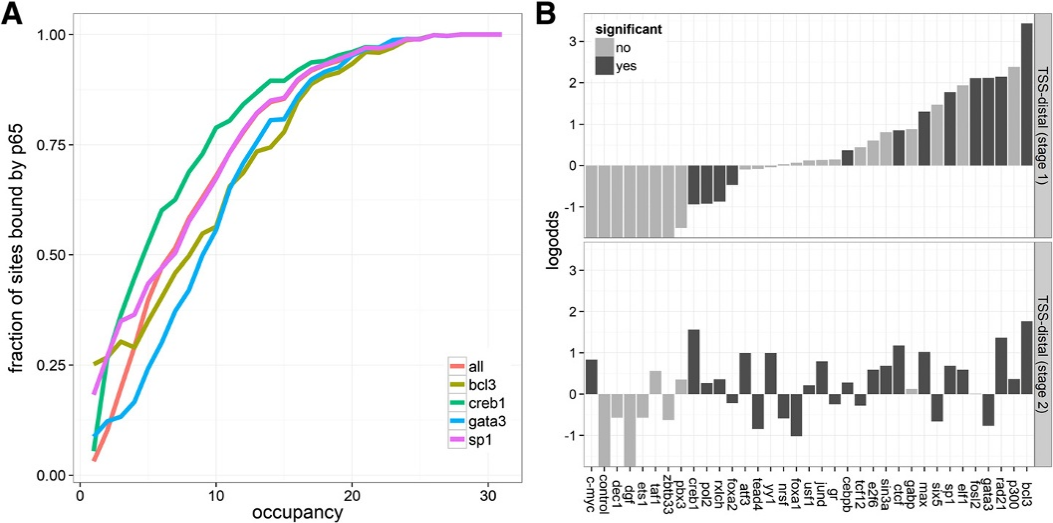
Role of pre-bound transcription factors in p65 recruitment. **a** Probability of p65 recruitment plotted as a function of the number of transcription factors (TFs) pre-bound at regulatory sites (occupancy). CTCF and RAD21 (cohesin) do not contribute to the occupancy count. **b** Enrichment of p65 binding events at two classes of regulatory sites. TSS-distal *stage 1* sites have a single TF pre-bound. TSS-distal *stage 2* sites have TF occupancy from 2 to 4. Log-odds of p65 recruitment reported for each protein with all *stage 1* and *stage 2* sites as background, respectively. Significance estimated by the Fisher’s exact test. TFs are ordered based on log-odds at *stage 1* TSS-distal sites

Our first goal was to understand the general trend by which chromatin-bound proteins influence p65 recruitment. Recent findings suggest that following stimulus the induced transcription factors frequently localize at “pre-coded” sites with a high-occupancy of other TFs (HOT regions) [37, 118]. We observed that the probability of p65 binding increases monotonically with the number of proteins present at a site (Fig. 3a). For example, sites which are bound by 12 proteins have a 75 % chance of recruiting p65 after stimulation. The presence of specific TFs can increase or decrease binding frequency. We found, among others, that sites bound by GATA3 and BCL3 are overall less likely to recruit p65, while sites bound by CREB1 have an increased propensity for p65 (Fig. 3a). Importantly, BCL3 is a nuclear IκB protein with strikingly opposite and poorly understood roles in the regulation of NF-κB binding and activity [119, 120].

#### Pre-bound TFs recruit p65 to specific regulatory regions (association analysis)

To quantify the strength of association we calculated the odds of p65-recruitment contingent on the presence of chromatin-associated proteins at TSS-distal regulatory regions (Fig. 3b). To control for occupancy we analyzed single-factor *stage 1* and medium-occupancy (2–4 pre-bound TFs) *stage 2* sites individually and evaluated statistical-significance using the Fisher’s exact test (see Methods). We found that BCL3, RAD21 (cohesin), and MAX, were most significantly associated with p65 recruitment at both classes of sites. Together, these observations show that pre-bound transcription factors influence the propensity of a site to recruit p65 after induction. More importantly, they suggest that sites co-occupied by several transcription factors can have markedly different propensities for p65 binding than the individual proteins, which provides indirect support for combinatorial regulation of transcription by specific TF recruitment [4, 88].

### Cooperative p65 recruitment by clusters of TFs

#### Use of a “cooperativity matrix” for the identification of recruitment synergism

**Fig. 4:**
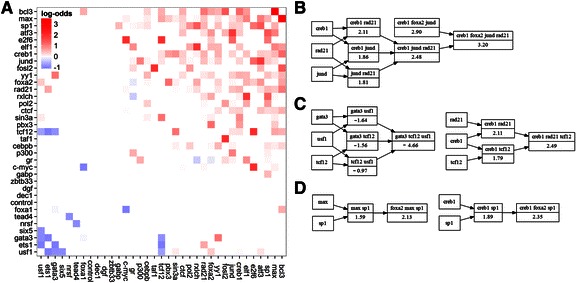
Cooperativity of transcription factors in p65 recruitment. **a** Matrix of positive (red) and negative (blue) cooperativity between transcription factors (TFs) in p65 recruitment. Positive (negative) cooperativity means that sites co-occupied by the two TFs have higher (lower) propensity for p65 than sites occupied by any of the TF alone. Only significant enrichments are shown *p* < 0.05. Enrichments that do not meet a stringent significance level *p* < 1e-5 are capped at log-odds 2 or −1, respectively. **b**–**d** Each diagrams represents a single “TF-cluster” obtained using the TGC algorithm (Methods). Each box represents TSS-distal *stage 2* sites co-occupied by the indicated transcription factors (TFs). Log-odds enrichment scores are indicated below the TF symbols and are all significant (*p* < 0.05). Arrows represent the intersection operation. Successive intersections within a single TF-cluster monotonously improve enrichment. **b** TF-cluster with the highest positive propensity for p65 recruitment **c** Context dependent role of TCF12. Together with GATA3 and USF1 TCF12 forms the only TF-cluster which is depleted of p65 binding events. Together with CREB1 and RAD21 it forms a TF-cluster with moderate positive cooperativity. **d** FOXA2 has weak propensity for p65 recruitment on its own, but boosts the propensity of some combinations of TFs

We decided to test this hypothesis computationally, with regard to p65 binding, by determining whether combinatorial interactions of proteins at the chromatin-level lead to cooperative recruitment of p65. We focused on *stage 2* enhancers, as these sample pairwise interactions exhaustively, while controlling for DNase I accessibility and occupancy number (see Methods). Importantly, less than 10 % of *stage 2* TSS-distal sites recruit p65 (see “all” TF curve with occupancy ranging from 2 to 4 in Fig. 3a), which suggests a relatively large degree of binding specificity. We calculated a “cooperativity matrix” (Methods), which shows whether sites co-occupied by pairs of TFs have increased (positive cooperativity) or decreased (negative cooperativity) propensity for p65 binding (Fig. 4a). A similar matrix was calculated for low-occupancy promoters (i.e. *stage 1* TSS-proximal sites). These results are similar, and support most of the cooperative interactions observed at *stage 2* enhancers (not shown).

#### Strong synergism between BCL3 and MAX in p65 recruitment

We found that the majority of interactions shows positive cooperativity; some of which are surprisingly strong. For example, sites occupied by both BCL3 and MAX show 37-fold higher propensity (log-odds 5.22) for p65 over sites bound by two random proteins (Methods). The synergism of this pair improves over the already strong enrichment of BCL3 on average (log-odds 1.77) (Fig. 3b). Several TFs participate in multiple synergistic interactions, most notably SP1, MAX, JUND, and YY1. Importantly, all of these TFs show individually a strong propensity for p65 (Fig. 3b). Several TFs appeared to form “cooperativity cliques”. For example, all pairwise interactions between MAX, RAD21, and JUND show positive cooperativity in p65 binding (Fig. 4a). Together, this suggests that p65 is recruited by clusters of chromatin-associated proteins with strong affinity for p65.

#### Association rules for the identification of cooperative TF clusters (optimization of combinations of TFs that recruit p65)

Our next goal was to identify combinatorial clusters of TFs that are predictive of p65 site selection [121, 122]. Intuitively, such “TF-clusters” should show the highest propensity for p65 when they are fully assembled on the chromatin. We used two heuristics that attempted to find orthogonal solutions to this combinatorial problem. The first method (TGC) was “greedy”, but optimized the statistical association (enrichment p-value) directly. The second technique (TNR [77]) attempted to identify all associations (strictly association rules [123]) with strong statistical support, which we next filtered for those that predict p65 binding (see Methods and Additional file 1: Methods). We ran both algorithms on TF occupancy data at *stage 2* enhancers and report on the most significant TGC clusters as these were a high-confidence subset of the TNR clusters.

#### Sites occupied by CREB1, RAD21, JUND, and FOXA2 are preferentially bound by p65

We found a total of 21 TF-clusters with a high propensity for p65, and one which was significantly depleted of p65 binding (Table 2). *Stage 2* enhancers occupied by any of these TF-clusters were on average 4-fold enriched for p65 and predicted p65 recruitment with genome-wide confidence (i.e. probability of p65 recruitment) over 60 % . Combinatorial clusters with the strongest enrichment had confidence over 70 % (Fig. 4b-d, Table 2). The TF-cluster with the highest propensity for p65 binding involved CREB1, RAD21, JUND, and FOXA2 (Fig. 4b). The only cluster with negative cooperativity in p65 recruitment comprised GATA3, TCF12, and USF1 (Fig. 4c left). Strikingly, TCF12 was also found in a positively cooperative TF-cluster with CREB1 and RAD21 (Fig. 4d right). Consistently, TCF12 was the only context-dependent TF involved in both negatively and positively cooperative pairwise interactions (Fig. 4a). On the contrary MAX and BCL3, which have high individual propensity did not appear to form cooperative clusters among the included TFs.

**Table 2:** Combinatorial clusters of chromatin-bound TFs most significantly associated with p65 recruitment

cluster	log-odds	*p*-value	supp.	conf.
creb1-foxa2-jund-rad21	3.20	1.4E-11	7890	0.76
creb1-rad21-usf1-yy1	2.56	1.4E-09	5815	0.67
foxa2-rad21-usf1-yy1	2.03	9.1E-07	5672	0.59
gata3-tcf12-usf1	−4.66	1.1E-08	12907	0.01
creb1-rad21-sp1	2.90	1.4E-26	9415	0.72
max-rad21-sp1	2.74	4.6E-15	10061	0.70
creb1-foxa2-yy1	2.52	1.7E-14	11814	0.67
creb1-max-rad21	2.49	3.6E-87	11088	0.65
creb1-rad21-tcf12	2.49	1.1E-11	8507	0.66
max-rad21-tcf12	2.44	2.6E-09	9096	0.65
creb1-foxa2-sp1	2.35	4.8E-10	18956	0.64
jund-max-rad21	2.24	6.3E-10	9186	0.62
foxa2-max-sp1	2.13	1.8E-06	20425	0.60
gr-max-rad21	2.11	2.8E-12	9346	0.60
jund-pol2-rad21	2.08	7.6E-06	6302	0.60
cebpb-jund-rad21	2.01	6.5E-07	6442	0.58
max-pol2-rad21	2.00	4.5E-17	7741	0.58
cebpb-foxa2-yy1	1.95	2.9E-05	7025	0.58
elf1-foxa2-rad21	1.71	1.9E-08	4304	0.53
foxa2-max-yy1	1.62	4.2E-05	11994	0.52
cebpb-foxa2-usf1	0.90	6.6E-05	12804	0.39

TF-clusters obtained by the TGC algorithm at *stage 2* TSS-distal sites. Each cluster is significantly enriched (or depleted) for p65 binding events. The enrichment log-odds and significance are from the Fisher’s exact test. Support (supp.) and confidence (conf.) are calculated genome-wide for all regulatory sites. Support is the number of sites occupied by all TFs from a TF-cluster, confidence is the percentage of these sites that will recruit p65

#### Cooperative clusters are consistent with the pioneer-constitutive-inducible sequence of enhancer assembly

According to the current model of sequential enhanceosome assembly [35, 37, 124], lineage-determining TFs, such as PU.1 (SPI1) initiate remodeling at tissue-specific enhancers and act as pioneer TFs, which have the ability to “open” closed chromatin, particularly at H2A.Z-containing nucleosomes [110]. These accessible sites are then bound by ubiquitous TFs, which facilitate selective access for stress-inducible TFs. Our results are consistent with this model. First, we observed that the “pioneer” TF FOXA2 was positively associated with p65 recruitment only at sites, which were further co-occupied by additional TFs. FOXA2’s ability to markedly increase p65 binding frequency appeared to require at least two other proteins (Fig. 4d). Conversely, ubiquitously expressed TFs – SP1, MAX, YY1, JUND – had intrinsic individual propensities for p65, which were boosted through their frequent pairwise and higher-order interactions (Fig. 3b, Fig. 4, Table 2). For example, the TF-cluster with the largest p65 binding odds contained the transcription factors FOXA2 and JUND, together with RAD21 and CREB1 (Fig. 4b). In agreement with the enhanceosome model FOXA2 is the pioneer, while JUND unlike other AP-1 family members is constitutively expressed [125]. AP-1 (JUND/FOSL1) and NF-κB (p65/p50) have been shown to be required for the induction of the IL6 gene [126]. Consistently, we found that IL6 is among the most up-regulated genes (23-fold). We found that the majority of TF-clusters, including the top FOXA2-JUND pair, further included CREB1, RAD21, or both (Table 2). It has been recently reported that both, RAD21 (cohesin) and CREB1, have a constitutive role in the stabilization of large protein complexes at tissue-specific loci [127, 128]. Together, these observations suggest that the layered TF-network (pioneer, ubiquitous, induced) [37] involves CREB1 and RAD21 as scaffolding components. Strikingly, SP1, YY1, and JUND, have been shown to physically interact with each other or NF-κB [129–131], and thus might confer specificity for p65.

We observe a similar TF-network at the negatively cooperative TF-cluster (GATA3, TCF12, USF1). Analogously, GATA3 is a pioneer TF [132], USF1 is an ubiquitously expressed basic helix-loop-helix (bHLH) transcription factor [133], while TCF12 (HEB) is a bHLH required specifically for T-cell development [134]. Further, all three proteins show negative cooperativity with ETS1, a pioneer factor from the same family as PU.1. Consistently, several pairwise interactions within a small subset of proteins, most notably ETS1 and GATA3, showed moderate negative cooperativity in p65 recruitment (Fig. 4a). Interestingly, ETS1 is also also typically restricted to the T-cell lineage, which when compared to PU.1, shows a remarkable difference in sequence specificity, and was shown to interact with *NFKB1* (p50) [40, 135, 136].

### p65-mediated induction of pre-assembled promoters

**Fig. 5:**
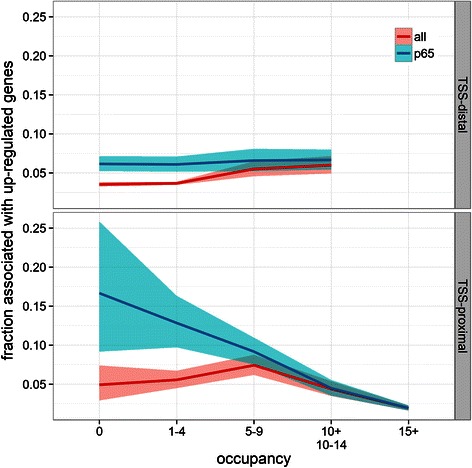
p65-mediated transcriptional activation. Each gene was linked to 1 TSS-proximal and at most 3 TSS-distal regulatory sites. The plot shows the fraction of sites linked to genes that will be up-regulated after p65 activation as a function of occupancy (see Fig. 3). Sites bound by (p65) are compared to (all) other sites

We attempted to identify TSS-proximal and TSS-distal sites that are most frequently associated with p65-upregulated genes [137]. We find that the probability of a p65-bound site to be associated with an up-regulated gene depends, not surprisingly, on whether the site is a promoter or enhancer (Fig. 5). Reassuringly, we observed, that among all profiled TFs, sites bound by p65 are up-regulated most frequently (proportion difference *p*-value: 0.001 [79]). This confirms the role of p65 as a transcriptional activator. In addition, of the 415 known NF-κB target genes (see Methods), 102 are differentially expressed with 89 upregulated. Moreover, 51 of these known NF-κB target genes are differentially expressed and contain a p65 peak in their promoter (Additional file 2: Data). Strikingly, we found that the ability of p65 to induce gene expression is much higher at low-occupancy p65-recruiting promoters (low-occupancy *vs* high-occupancy *p*-value: < 1e-4), whereas no strong influence of occupancy on p65-induction was seen at TSS-distal sites (Fig. 5). This re-affirms that p65 binding to inaccessible sites (Fig. 1), is “functional” and has on average the same impact on transcriptional activation as p65 recruitment to pre-accessible sites (approx. 6 %). It also suggests that a relatively large subset (approx. 16 %) of low-occupancy promoters are particularly susceptible to transcriptional activation. As a corollary genes with high-occupancy promoters are not easily inducible (< 5 %). We observed that low-occupancy promoters bound by TAF1 had the highest chance of being up-regulated (Additional file 1: Discussion, Additional file 1: Figures S7-9), which raises the possibility that specific TFs loaded at TSS-proximal or TSS-distal sites would bias genes towards p65-mediated activation.

## Discussion

After treatment of A549 with TNF and subsequent NF-κB activation we observed 65,253 p65 (RELA) binding sites genome-wide (Fig. 1). We tried to determine genome-wide cues that guide p65 to select accessible sites or alternatively bind at ostensibly inaccessible regions. Paradoxically, p65 binding was so ubiquitous that the majority of genes had a p65 peak in their proximity (not shown), which is in stark contrast to the relatively small number of 716 genes that were differentially expressed.

Our goal was to understand the regulatory logic of the NF-κB transcriptional network. Through large-scale data-mining and the development of *sensu stricto* predictive models we identified a number of testable hypotheses regarding which genomic features influence p65 binding site selection, and which promote p65-mediated transcriptional activation. We have found evidence of rapid p65 binding outside accessible chromatin; importantly this type of remodeling activity is typically attributed to pioneer transcription factors. Further, we identified combinatorial patterns of epigenetic marks and transcription factors that predict p65 recruitment. This type of association, although not strictly causal, shows to what extent the induced p65 cistrome is predetermined by the extant chromatin landscape.

### NF-κB is recruited to chromatin like a pioneer TF

NF-κB dimers are believed to fall into the category of “signal sensors” inducible transcription factors (TF) that opportunistically exploit a pre-existing enhancer landscape [15, 40]. Our analyses suggest that p65 is recruited to all types of enhancers including “latent”, “primed”, and “active” ones [48, 93, 97, 102]. Our results suggests that within 30 min of TNF stimulation p65 is already, albeit at a relatively low level (Additional file 1: Figure S3), recruited to 16,032 putative latent enhancers in A549 cells, which represent 24.5 % of all p65 binding. This is similar to the time frame required for the “pioneer” transcription factor PU.1 to bind latent enhancers in macrophages (Fig. 4 in [93]). Consistently, Jin et al. have shown that in HeLa cells 17 % of p65 binding occurs outside accessible chromatin (Fig. 2d in [15]). These numbers should be contrasted with, for example, *GR* and *FOXP3*, which have, 5 and 2 % sites outside DHS regions, respectively [138, 139]. On the other hand, it has been previously shown that cytokine induced TFs have the ability to activate lineage-specific enhancers. A recent report suggests that gains in H3K4me1 and p300 at a subset of enhancers in Th2 cells are mediated by *STAT6* [48].

Strikingly, we observed that p65 binding outside of CpG islands (CGI) occurs both at hyper- and hypo-methylated CpG sites (Fig. 1d). It has been recently shown that a similar subset of non-CGI regions switch from DNA methylation to H3K27me3 during early embryonic differentiation [92]. This process appeared to be associated with *FOXA1* and its quite unique ability to bind methylated DNA and initiate chromatin remodeling [29, 91]. What is more, *FOXA1* and *FOXA2* have been shown to bind nucleosome-wrapped DNA [27, 110], and similarly, NF-κB dimers have a demonstrated ability to recognize nucleosome-occluded κB elements [140]. Pioneer TFs have the unique capability to trigger cellular reprogramming while chromatin remodeling was shown to be an early bottleneck of this process [31–33] (reviewed in [24, 34, 141]). Likewise, induction of NF-κB activity has been shown to accelerate epigenetic reprogramming, the epithelial-mesenchymal transition, and dedifferentiation [49, 51, 142]. Interestingly NF-κB activity is also required for B-cell and T-cell development and maturation [8, 143]. Since there is no evidence that NF-κB has the ability to initiate chromatin remodeling (reviewed in [40]) it is likely that the the above analogies reflect synergism between NF-κB and some unknown constitutive or induced pioneer transcription TF(s); possibly from the Stat or Irf families, which have overlapping targets and similar roles in development and cancer (e.g. [144, 145]).

### NF-κB induces expression from enhancers and low-occupancy promoters

Further, our results highlight that p65 recruitment to latent enhancers has similar influence on the induction of putative late response (not simply binding) to primed and active regulatory regions (Fig. 5), which suggest that during the course of TNF treatment some of the latent sites ultimately develop into high-occupancy enhancers, recruit additional co-activators, and enhance transcription. Strikingly, we have found that promoters of the most upregulated putative late response genes had low-occupancy promoters with a *RNAPII* positioned immediately downstream of the TSS (Additional file 1: Figure S8), and conversely that at high-occupancy promoters p65 binding had less impact on transcription. Genes with a fully “loaded” preinitiation complex (PIC) including TFIID (*TAF1*) and other co-activators were modestly upregulated [146]. Why the majority of p65-bound promoters are inefficiently or transiently induced is, however, still an open question.

### Epigenetic codes predict p65 recruitment

We observed that within accessible sites epigenetic marks and their combinatorial patterns are quantitative predictors of p65 recruitment. Building on initial results from previous reports [94, 147, 148] we found that marks influence the selection of binding sites even after controlling for DNase I hypersensitivity (DHS). Importantly, in the previous studies TF peaks and DHS sites were mapped in the same cellular state and provide limited independent information as they are expected to correlate almost perfectly [81]. We also found that overall H3K4me1 and H2A.Z, a histone variant associated with high nucleosome-turnover [149], are most significantly associated with p65 binding at enhancers and promoters (Fig. 2c–d). Importantly, H3K4me1 deposition has been shown to be triggered by lineage-specifying TFs [47], and not to change in response to stimulus [35, 89] (but see [48, 93]), and interpreted to mark enhancers “primed” for activation [97]. Our results suggest that H3K4me1 strongly influences p65 binding probability (Table 1) and provide evidence for synergism and context-dependent roles of select combinations of histone modifications in the recruitment of p65 judging by the uninterpretable results of simple additive models (Additional file 1: Figure S10). We find, for example, that H2A.Z is involved with other histone modifications in the cooperative recruitment of p65 (Fig. 2, Additional file 1: Figure S3) and that the co-occurrence of H3K4me1 and H2A.Z is the strongest predictor of p65 binding to TSS-distal enhancers and hence a *de facto* signature of “primed” enhancers [101]. Strikingly, we found that H2A.Z can have the opposite effect on p65 binding when it is in another context with the repressive H3K27me3 methylation (Fig. 2c). These findings are in agreement with a recent study which showed that recruitment of PRC2, the complex that deposits H3K27me3, is dependent on H2A.Z [115]. Interestingly, H2A.Z removal can be triggered by the pioneer TF FOXA2 [110].

### Recruitment of p65 by pre-bound transcription factors

Current models of stimulus-dependent transcriptional activation describe the sequential recruitment of increasingly specialized TFs which leads to gene expression [35]. In general terms, competence for induction by the most specialized TF (including NF-κB) was proposed to depend on the combination of previously recruited proteins [37]. Implications from this model prompted us to explore whether pre-bound TFs influence p65 binding, and whether sites co-occupied by clusters of several TFs showed cooperativity in p65 recruitment. We found that sites occupied by BCL3 showed the largest propensity for p65. BCL3 is a nuclear IκB protein, but its role in the regulation of NF-κB binding and activity is context-dependent and poorly understood [150–152]. Importantly, it has been shown that BCL3 and IRF3 might play complementary roles as co-activators, at a subset of NF-κB targets, in TNF- and LPS-induced cells, respectively [5], which could mean that BCL3 can initiate chromatin remodeling.

#### Enhanceosome assembly, p65 recruitment and clusters of cooperative TFs

Strikingly, we found that the role of multiple TFs is context-dependent. For example, CREB1 is negatively associated with p65 binding when present at a site where no other TF is bound, but shows positive cooperativity with several other TFs at co-occupied sites (Fig. 4a). One of the roles of CREB1 is to recruit CBP, which is both a histone acetyltransferase and protein scaffold [153]. Interestingly, several of the TFs engaged in cooperative p65 recruitment are known to physically interact with CBP, but not with each other. This observation, together with recent reports on the role of CREB1 and RAD21 (cohesin) in the stabilization of large protein complexes [127, 128], suggest that p65 binding at tissue-specific loci is stabilized by RAD21 or CREB1.

Finally, we have found that sites occupied by specific clusters of TFs have very large positive or negative propensities for p65 recruitment. In line with other studies we interpret this as traces of cooperative binding [117, 154]. However, direct interaction between the pre-bound proteins and/or p65 might not be strictly necessary as cooperative binding has been observed in vivo within larger and more complex regulatory sites [80, 154] and can arise from competition with nucleosomes [155, 156]. Importantly, the hierarchy of both TF-clusters is consistent with the current models of sequential enhanceosome assembly and combinatorial regulation of gene expression [116]. Sites occupied by (CREB1, RAD21, JUND, FOXA2), were most enriched for p65 binding, whereas those occupied by (GATA3, TCF12, USF1), were most depleted. Interestingly, AP-1 (JUN/FOS) is also involved in the maintenance of glucocorticoid receptor (GR) accessible sites [22]. GR has an important anti-inflammatory role and acts *via* the inhibition of NF-κB-mediated gene induction [157, 158]. This suggests that NF-κB and GR have overlapping sets of target sites. A striking example is the transcriptional regulation of the IL6 promoter. Induction of IL6 has been shown to depend on p65 and JUND recruitment, whereas silencing required GR binding, which was mediated by FOXA2 [159].

## Additional files

Additional file 1: **Supplemental Material (Discussion, Methods, Figures, and Tables).** (PDF 2793 kb) Additional file 2: **NF-κB Target Genes (Gene Name, RefSeq Id, Ensembl Id), log**_**2**_**Fold Change (TNF stimulated vs unstimulated), Differential Expression Significance Call (TRUE/FALSE) and NF-κB Peak Detected after TNF Stimulation (TRUE/FALSE) (Data).** (CSV 24 kb)
