# Supplementary material for: Genome-wide predictors of NF-κB recruitment and transcriptional activity
Source: BioData Min. 2015 Nov 26;8:37. doi: 10.1186/s13040-015-0071-3 (PMC4661973; doi:10.1186/s13040-015-0071-3)
Supplement: Additional file 1: — Supplemental Material (Discussion, Methods, Figures, and Tables). (PDF 2793 kb) [file 13040_2015_71_MOESM1_ESM.pdf]

Supplementary Discussion

Synergism between H2A.Z and histone acetylations in p65 recruitment

To detect synergistic effects between epigenetic marks we used two orthogonal approaches. The first method relied on differences in correlations between epigenetic marks at p65-bound and unbound sites. Intuitively, if two marks show synergism in recruiting p65, they ought to be more correlated at p65-bound sites. Conversely, if p65 selects sites with high levels of either modification (but not both), these marks ought to be less correlated. This comparison revealed two general trends (Figure S5A). At p65-bound stage 1 enhancers permissive histone modifications were more correlated with each other, while at p65-bound promoters they were de-correlated from H3K4me1. To understand the cause of these effects better, we compared H3K4me1 and H3K9ac levels at p65-bound and control regions (Figure S3). Strikingly, we found that at stage 1 enhancers (Figure S3B) these marks were strongly correlated, whereas at promoters (Figure S3A) they were mutually exclusive. We found that at TSS-distal sites H3K9ac enrichment correlated with and was contingent on H3K4me1 presence. We found 12,682 sites that were enriched (scaled level > 0.1) for H3K4me1 with no H3K9kac (scaled level < 0.03), but only 1,534 sites with H3K9ac without any H3K4me1 according to identical criteria. Of the 6,894 sites without any H3K4me1 only 11 were bound by p65 (Figure S3B). At promoters, however, some level of both marks was required for p65 binding (Figure S3A). In particular sites without H3K9ac were 6-times less likely to recruit p65 than sites with even minimal levels of this mark (not shown).

Next, we have developed a statistical method to directly test which histone modification pairs show positively or negatively cooperative effects on p65 recruitment (see Methods). In simple terms we identify pairs where elevated levels of two marks are associated with p65 more significantly than any of the marks individually. We found that, overall, cooperative effects were rare and relatively weak, but most importantly different between TSS-proximal and TSS-distal sites (Figure S5B). Strikingly, the only cooperative interaction, common to all three classes, was between H3K4me2 and H2A.Z. At promoters we observed positive cooperativity of H3K4me1 with classic activating marks, most prominently H3K9ac, but there was no evidence for cooperativity between H3K4me1 and active marks at *stage 1* enhancers. In contrast, the majority of cooperative interactions at *stage 1* enhancers involved H2A.Z (Figure S5B bottom). The strongest cooperative effect was observed for H2A.Z and H3K9ac, with further synergies between H2A.Z and other classic activating modifications (H3K4me3, H3K27ac). It is noteworthy that in that regard *stage 2* enhancers are more similar to promoters than *stage 1* enhancers. In particular, marks at promoters and *stage 2* enhancers are similarly correlated (Figure S6A) and show quite similar cooperativity (Figure S5B).

p65-mediated induction of pre-assembled promoters (supplement)

We observed that low-occupancy promoters bound by several chromatin-associated proteins including *FOSL2*, *TAF1*, and *SP1* were upregulated with increased frequency (Figure S7). Promoters bound by TAF1 (TAFII250), AP-1 (FOSL2, JUND), and to a lesser extent SP1, CEBPB, and RAD21 were frequently over-expressed. Interestingly, TAF1 is the core scaffold of TFIID and part of the RNAPII preinitiation complex (PIC), cohesin has been shown to promote RNAPII pause-release [1], while both, AP-1 and SP1 physically interact with TFIID [2]. To investigate this further we examined the positioning of TAF1, RNAPII, and p65 at promoters of differentially expressed genes (Figure S8). We found that upon induction p65 bound within a region the size of one nucleosome upstream of the TSS, whereas RNAPII and TAF1 were pre-loaded immediately downstream. Both, RNAPII and TAF1 were detected at the majority of differentially expressed loci, but genes that were most highly upregulated (3-fold) appeared to bind TAF1 less frequently. Next, we looked into promoter occupancy to determine whether other co-activators or co-repressors were also present at these sites (Figure 8B). We found that the most upregulated genes had promoters with significantly lower occupancy, compared to both weakly induced and non-induced (Figure S9) genes. Together, these results show that transcriptional activation depends on both p65 recruitment directly upstream of the TSS and PIC pre-assembly. Most efficient induction is associated with low-occupancy promoters, while promoters associated with TAF1 are highly occupied with a median of 23 other TFs bound and over-expressed generally at a lower level. It is plausible that high-occupancy promoters correspond to immediate early genes (reviewed in [3]), given that their relatively modest over-expression was measured 12 hours after induction. On the other hand low-occupancy might have a slower activation kinetics due to the necessity to recruit several subunits of the polymerase holoenzyme.

Supplementary Methods

Combinatorial chromatin clusters of transcription factors (extended)

Top-k non-redundant rule (TNR) algorithm: TNR is an association rule mining algorithm [4]. It is an approximate algorithm and promises to retrieve non-redundant rules, which might not be strictly top-*k*. The algorithm takes multiple parameters, most importantly *k* – the number of associations to find, and *conf* the minimum association rule confidence. We used the implementation of TNR in the SPMF toolkit [5] and ran the algorithm with *k=400*, *conf=0.5*, and *Δ=100* on *stage 2* TSS-distal regulatory sites. The full output contains all association rules, but we are only interested in those, where p65 is the single *consequent i.e.* where p65 binding is predicted by the presence of a combination of TFs. All remaining rules were filtered. The confidence of the TNR output can be interpreted as a conditional probability:

$$P\left(p65 \mid \cap TFs\right)$$

The greedy complexer (TGC) algorithm: We have developed TGC to find combinatorial TF patterns (putative complexes) that are more strongly associated with p65 recruitment than the TFs individually. Like the TNR algorithm TGC attempts to find association rules with p65 as the *consequent*, but unlike TNR it is 1) biased toward discovering only this type of rules, 2) measures the strength of association via a statistical test (Fisher's exact test, FET) that directly measures the significance of an association, and 3) uses a simple, greedy optimization algorithm. We try to find TFs which are together (intersection) more strongly associated with p65 recruitment than the TFs individually. We use the FET to calculate the p-value of the intersection between 65-recruited accessible sites and TF-bound accessible sites. We identify complexes by taking the intersection of their binding sites and calculating the overlap p-value between p65-recruited sites and this combinatorial TF intersection. The algorithm maintains a list *C* of putative complexes *c*, which is initialized with individual TFs. The list is scanned for pairs of complexes *ci* and *cj*, whose intersection *cij* is more strongly associated with p65 than either *ci* or *cj*. Among these candidate pairs a single best pair is chosen and appended as a new "complex" to the list *C*. The scan is repeated for the elongated *C*, (ignoring the previously found pair). The algorithm terminates when no new pair can be found.

Differential Expression (extended)

For each sample `htseq-count` with default parameters was used to count RNA-seq reads within GENCODE exons. DESeq [6] coupled with FDR-corrected p-values gives a very conservative estimate of statistical significance, especially for experiments with a small number of replicates (in our case two). The number of protein-coding genes that met this stringent criterion was too low for analyses of genome-wide enrichment. To alleviate this problem, we decided to include all genes that had a nominal p-value of $p<0.026$, although these genes do not meet strict genome-wide significance they still represent the most up-regulated genes from the experiment.

Linking genes, TSS-proximal and TSS-distal sites (extended)

TSS-distal regulatory sites are putative enhancers (short E), however without special data it is impossible to definitely indicate which, if any, TSS-proximal promoters (short P) they regulate. To create a reasonable set of links between putative enhancers and annotated promoters we follow an approach that integrates both, distance constraints, and the distribution of TFs at enhancers and promoters. To calculate their similarity in terms of TF occupancy we follow a probabilistic approach. Intuitively, an E-P pair is similar if it is bound by many of the same TFs, and even more so, if a putative enhancer is bound by promoter specific TFs (or *vice-versa*). To estimate the specificity of TFs at enhancers or promoters we calculate their frequencies at Es and Ps, respectively. However, the background frequency of TFs depends on the occupancy of a site. For example, at enhancers with low-occupancy FOXA1 is relatively more frequent than at high-occupancy Es or Ps. Therefore, we calculate background frequencies for each occupancy-count

individually *i.e.* we obtain 72 background frequency vectors – one for each of the 36 possible occupancy-counts, independently for E and P. This allows us to calculate a similarity between two sites.

Intuitively, we calculate a score based on the joint probability of two binary vectors. We assume independence, between the vectors and between all marks. First, for each TF we calculate the probability of the observed joint-outcome. Possible joint outcomes are: TF bound in E - TF bound in P; not bound in E - not bound in P; not bound in E - bound in P; bound in E - not bound in P. To calculate this probability we use the background frequency of the TF at sites of the same class (E or P) and occupancy. Next, we transform these probabilities into *log10* scores, with a positive signs if the two sites "match" (*i.e.* both are bound (or unbound)), or a negative sign, if they don't. Finally, assuming independence, we sum the positive and negative (log-odds) TF-scores into a single vector-pair-score (E-P-score). The higher the score the more similar the two vectors and the less likely the similarity is by chance. Having defined a probabilistic E-P score we can use it to link the most likely E-P pairs.

Specifically, we link enhancers to promoters. For each P we scan a 200kb region centered on the P site and locate the 10 closest E sites. We keep 3 pairs (E-P links) with the highest score. Our next goal is to link TSS-proximal and TSS-distal accessible sites to canonical GENCODE v14 protein-coding genes. If a TSS-proximal site overlaps the TSS of a canonical protein-coding transcript it is assigned to this protein-coding gene. If a TSS-distal site is linked to a TSS-proximal site (E-P pair) it is assigned to the same gene as the TSS-proximal site. However, if the enhancer is not linked to any TSS-proximal site it is linked to the 3 closest genes within a 100kb window.

Combinatorial NMF "codes" of epigenetic marks (extended)

We have developed a method to decompose correlated levels of modifications into additive epigenetic "codes" using non-negative matrix factorization [7] and [Cieslik, submitted]. Briefly, each code is a sparse combination of correlated epigenetic marks. Within each code levels of marks are tied. The relative importance of a mark is proportional to its value (also called loading) within a code. Importantly, it is possible that marks captured by a code are correlated only within a sub-population of sites, or in other words codes are not required to be globally applicable. Intuitively, the method transforms locus-specific levels of individual marks into a small number of codes and their locus-specific weights. These weights correspond to the importance of each code at each site. The locus-specific levels of individual marks can be reconstructed from the prototypical codes and their locus-specific weights, however the reconstruction has an inherent error. The NMF algorithm attempts to find weights and codes that minimize the reconstruction error globally for all sites [8]. In summary, we obtain an additive parts-based representation of "chromatin patterns".

We benchmarked the code-based method against multivariate additive and combinatorial logistic regression model (Table S1). Individual models have been fitted on the three classes of regulatory sites (TSS-proximal, stage 1 and 2 TSS-distal). The three multivariate models include levels of all epigenetic marks (8) and the binary indicator of p65 motif presence as independent variables. The three combinatorial models include the above individual marks together with a small number of interactions. The interactions were limited to pairs that showed non-zero cooperativity. We found that all three types of predictive models have virtually the same performance and all are overall better than any of the univariate models (Table 1). However, in line with previous findings, the improvement which resulted from including multiple epigenetic marks was modest. It should be noted that the models differ in the number of free parameters. At the extreme, the cooperative mark-based model for *stage 1* enhancers has 18 terms while the code-based model has always only 4.

All logistic regression models ultimately link levels of histone modifications to a change in the log-odds of p65 binding. However, strong collinearity between the mark levels makes the coefficients of regression models difficult to interpret. This is obviously the case for the cooperative models where interacting terms are correlated to individual terms, but even individual marks are sometimes very highly correlated ($r > 0.8$). An example of this effect is presented in (Figure S6A). On the other hand decomposed NMF “codes” are less correlated ($r < 0.6$, Figure S6B), which is well below the rule-of-thumb “problematic” cutoff ($r > 0.7$). As a consequence we are more confident that the resulting code-based models quantitatively links combinatorial codes with p65 recruitment. High multicollinearity between epigenetic marks makes it difficult to estimate variable importance. For example, standardized slopes of the TSS-distal *stage 2* multivariate additive model ostensibly indicate that H3K9ac and H3K27ac have opposing roles in p65 recruitment. This is very likely an artifact since these two marks are very highly correlated and should not be included in a single model.

TABLES AND FIGURES

Table S1: Performance of multivariate models in the prediction of p65 binding

region	model	par#	AUC	MCC	prec.	rec.	F1
	cooperative	12+1	0.83	0.41	0.84	0.82	0.83
Prox	additive	8+1	0.83	0.40	0.84	0.85	0.85
	code-based	4+1	0.82	0.42	0.84	0.84	0.84
	cooperative	17+1	0.87	0.34	0.98	0.97	0.97
Dist S1	additive	8+1	0.86	0.33	0.98	0.96	0.97
	code-based	4+1	0.86	0.32	0.98	0.96	0.97
	cooperative	15+1	0.72	0.33	0.75	0.76	0.75
Dist S2	additive	8+1	0.72	0.32	0.74	0.77	0.75
	code-based	4+1	0.71	0.31	0.74	0.77	0.75

Predictive performance of multivariate: code-based, cooperative, and additive logistic regression models (see Supplementary Methods). Par# – number of model parameters; AUC – area under curve; prec. – precision; rec. – recall; F1 – F1 score. (+1) indicates that all models include the NFkB motif presence as a predictor.

Figure S1: *de novo* motif discovery at p65 summits

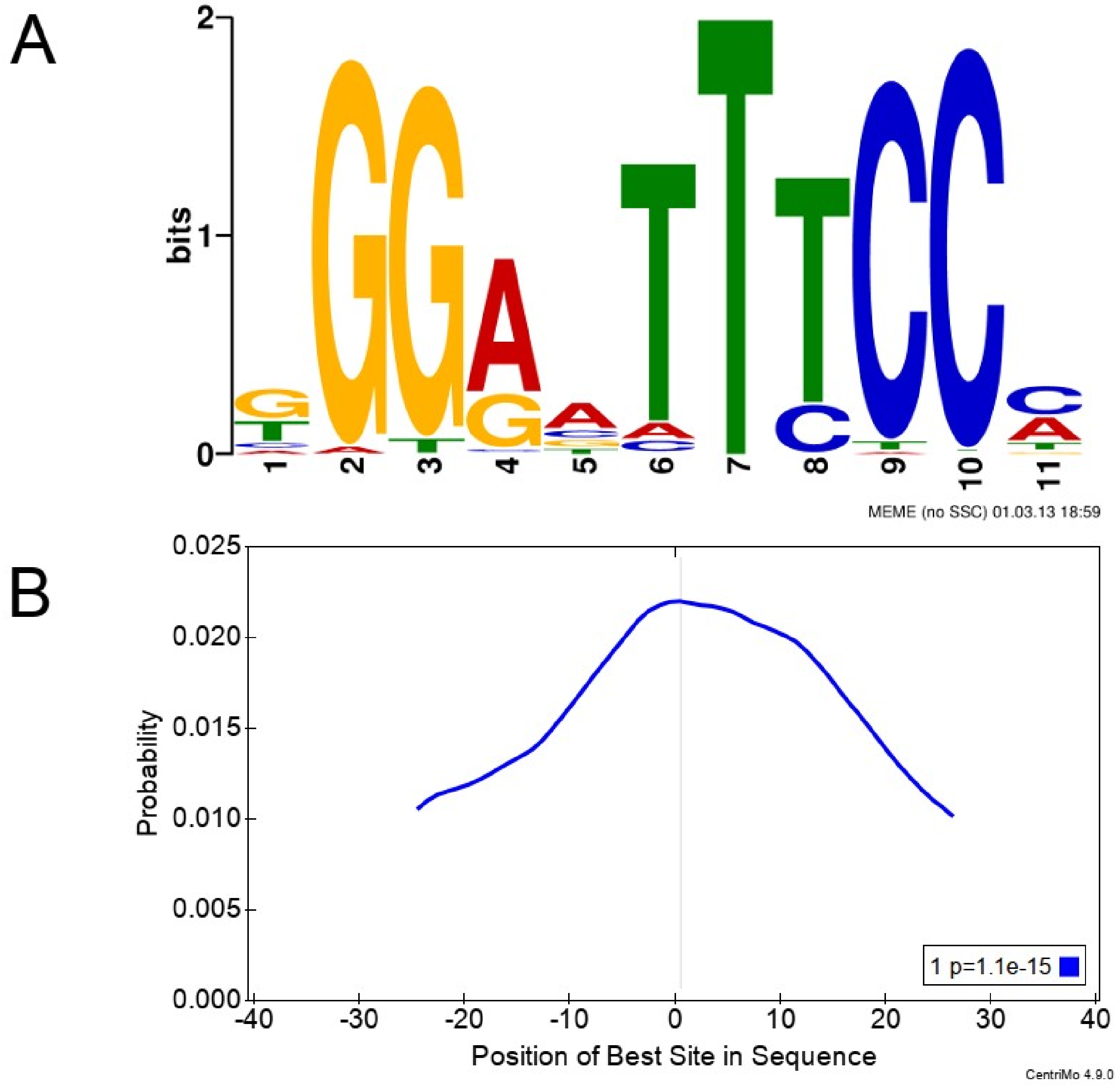

(A) Sequence logo of the only motif discovered *de novo* among sequences of 80bp windows centered on p65 summits outside of DNase I hypersensitive sites and regions bound by other transcription factors. The motif matches the consensus κB sequence (B) observed motif position relative to the p65 peak summit.

Figure S2: Distribution of epigenetic marks at p65 summits within *stage 2* TSS-distal regulatory sites

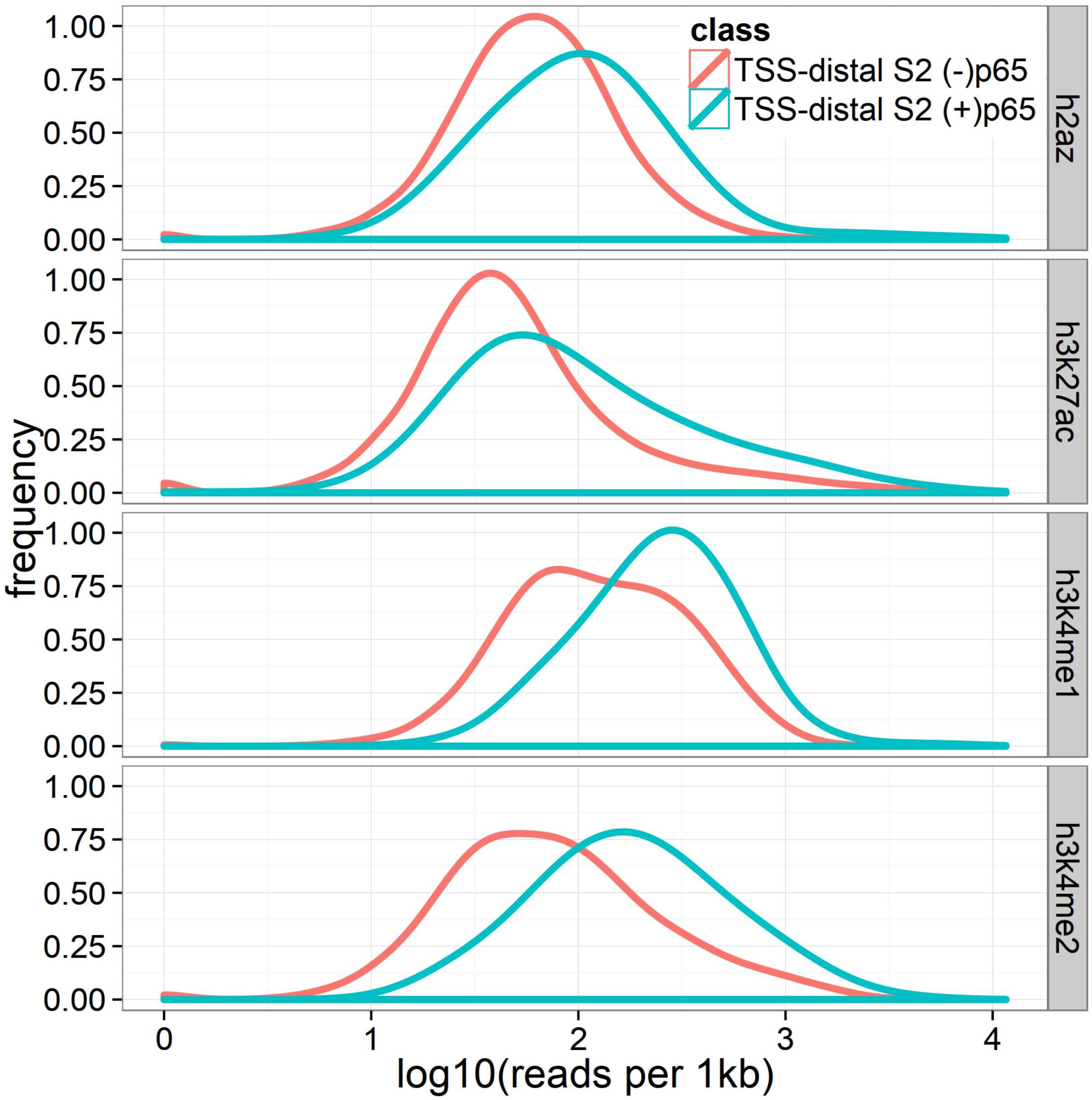

Densities of epigenetic marks at p65 peaks within accessible *stage 2* (S2) TSS-distal regulatory sites, bound by p65 (+) or not (-) (see Methods)

Figure S3: Enrichment profiles at p65 summits

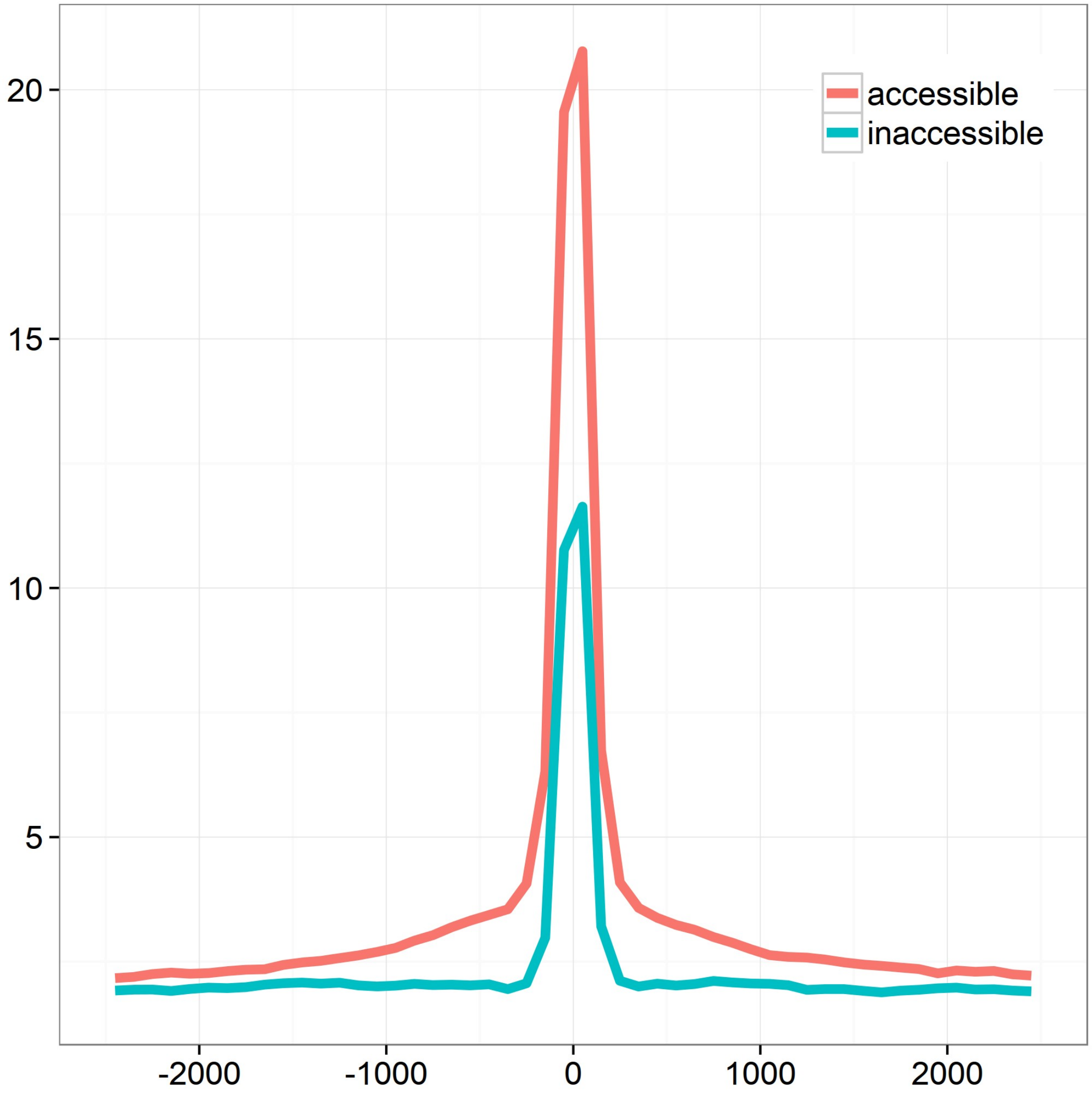

Mean enrichment profiles at p65 summits within accessible and inaccessible sites.

Figure S4: Differential correlation of H3K4me1 and H3K9ac

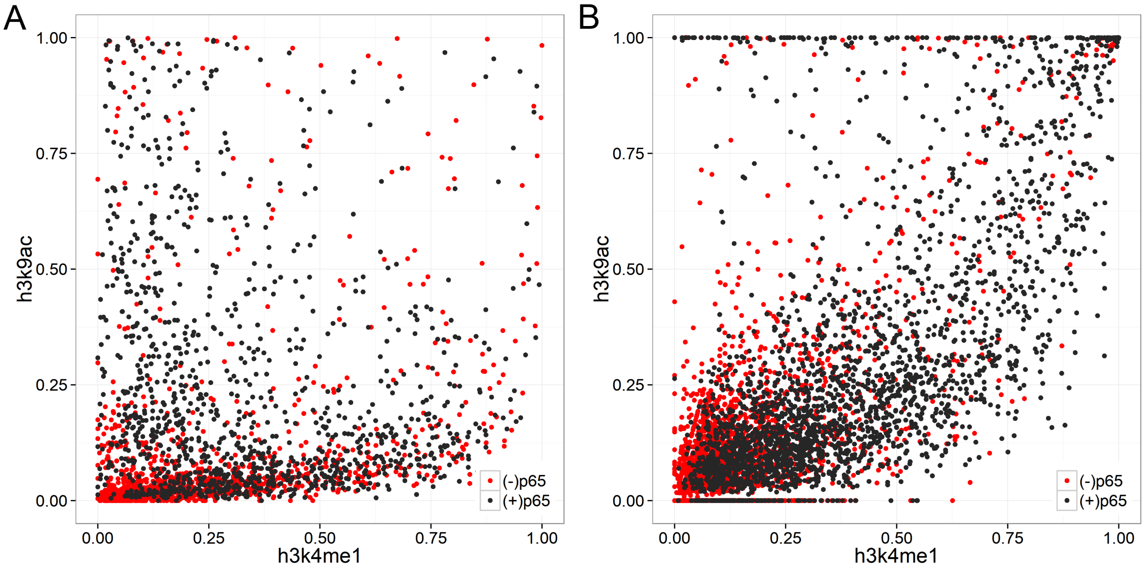

Each point represents a regulatory region. (A) TSS-proximal sites (B) *stage 1* TSS-distal sites. Sites that will recruit p65 are (black) and indicated (+)p65. Axes correspond to scaled levels of H3K4me1 and H3K9ac. Epigenetic mark levels are scaled individually for the two classes of regulatory sites (see Methods).

Figure S5: Cooperativity of epigenetic marks in p65 recruitment

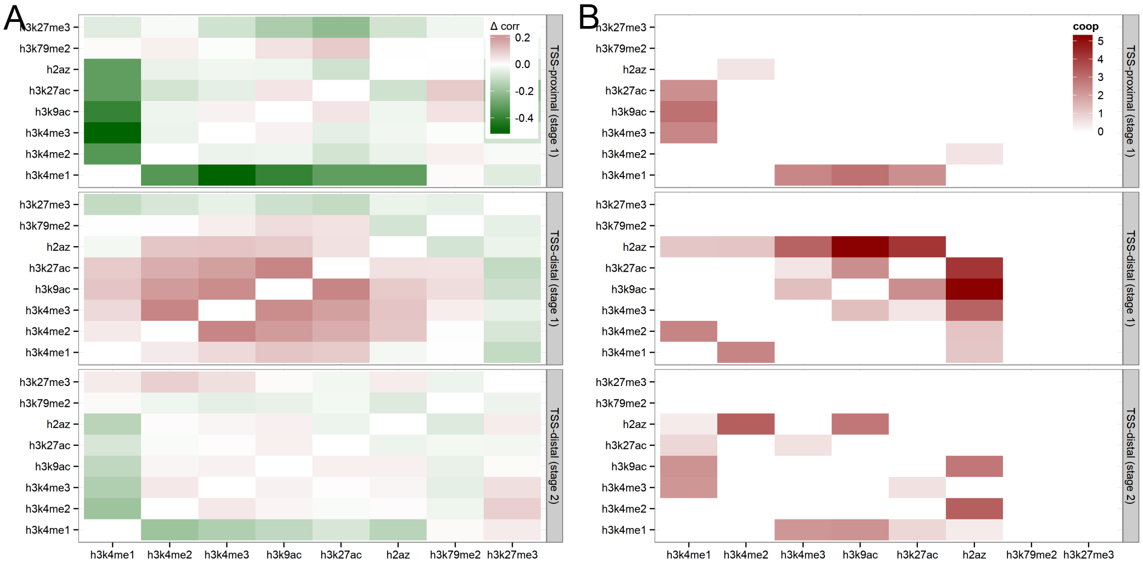

Assessment of cooperativity in the recruitment of p65 between pairs of epigenetic marks. Two methods have been applied within TSS-proximal and *stage 1* / *stage 2* TSS-distal regulatory sites. (A) Correlation difference heatmap. For each pair of epigenetic marks correlation was calculated at sites that will recruit p65 and sites that will not. The heatmap shows the difference in these correlations; positive values correspond to pairs that are more correlated at sites that will recruit p65. (B) Random-set cooperativity heatmap. Direct measure of pairwise cooperativity in p65 recruitment based on the random-set method. For each pair of marks their joint level was calculated using the geometric mean of their individual levels. Large cooperative values correspond to pairs whose joint level is more significantly enriched for p65 binding events than individual levels (see Methods).

Figure S6: Correlations between epigenetic marks and between codes

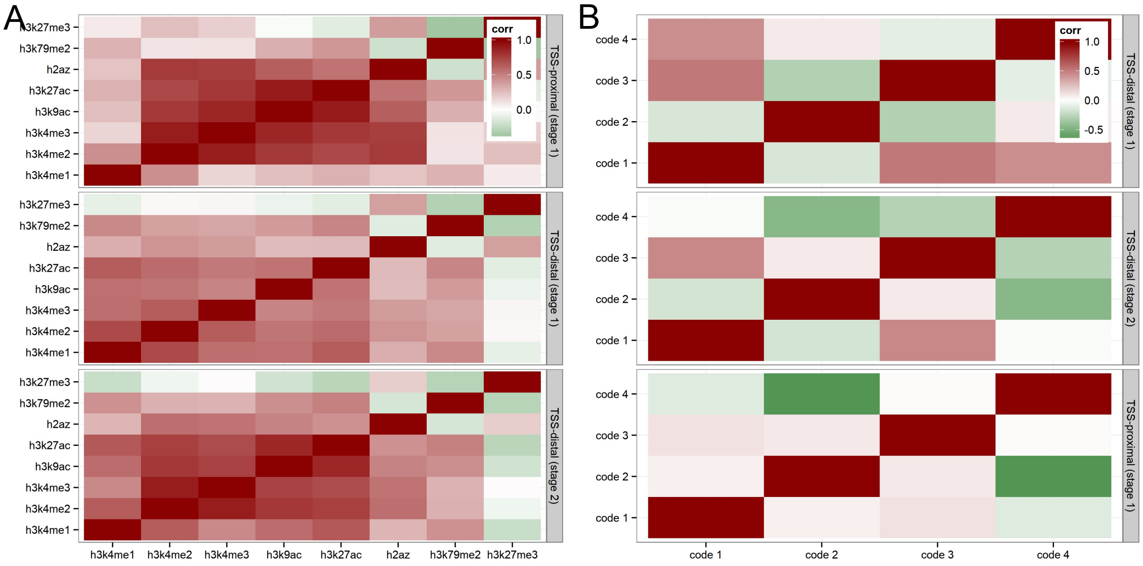

Heatmaps of Spearman's rank correlation coefficients (corr) between pairs epigenetic marks or codes calculated at three classes of regulatory sites. (A) correlations between epigenetic marks (B) correlations between epigenetic codes (see Methods).

Figure S7: Role of pre-bound TFs in transcriptional activation

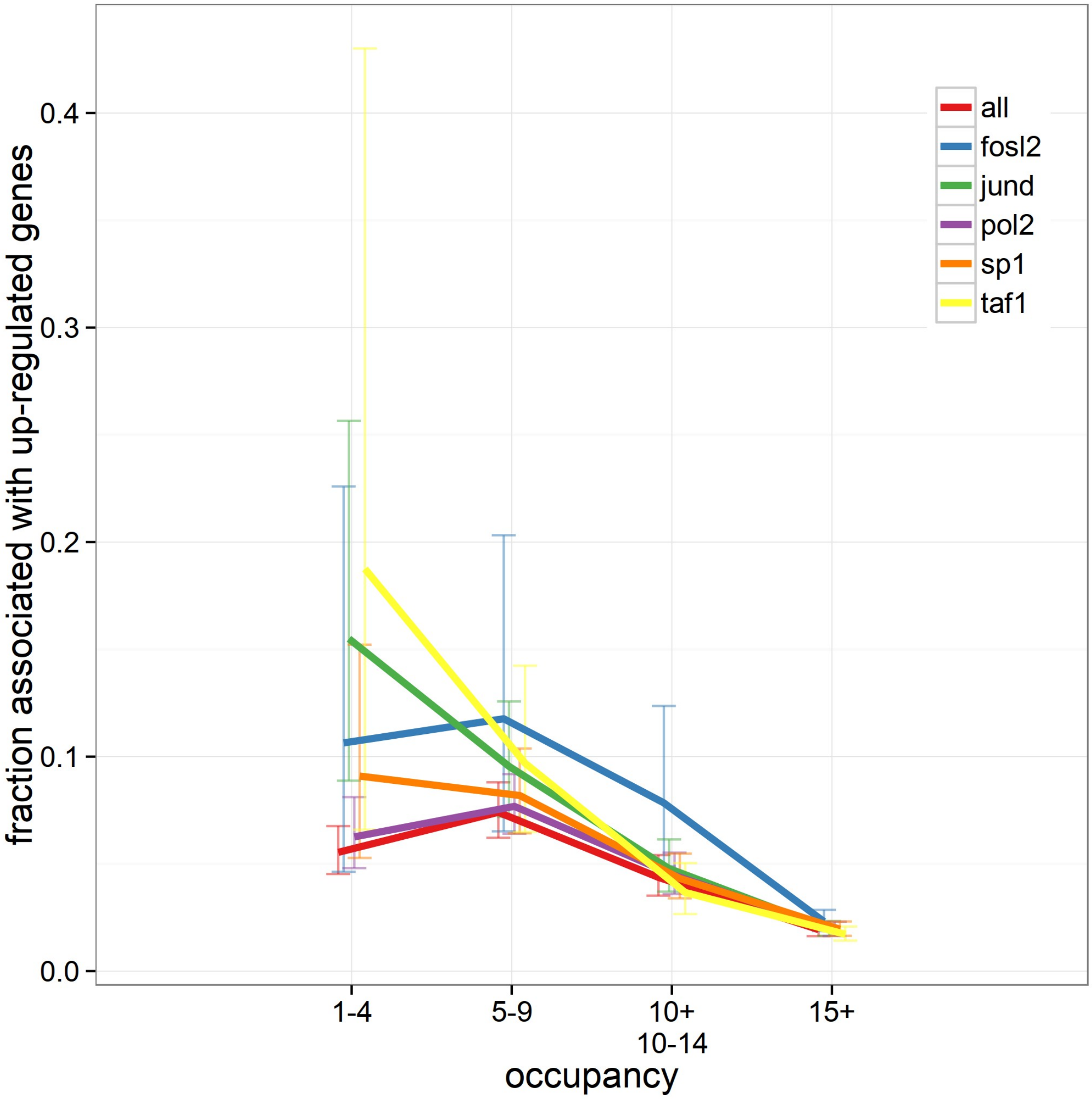

Each gene was linked to 1 TSS-proximal and at most 3 TSS-distal regulatory sites. The plot shows the fraction of sites linked to genes that will be up-regulated after p65 activation as a function of occupancy (see Figure 5). Sites bound by select TFs (color coded) are compared to (all) other sites.

Figure S8: Promoters of differentially expressed genes

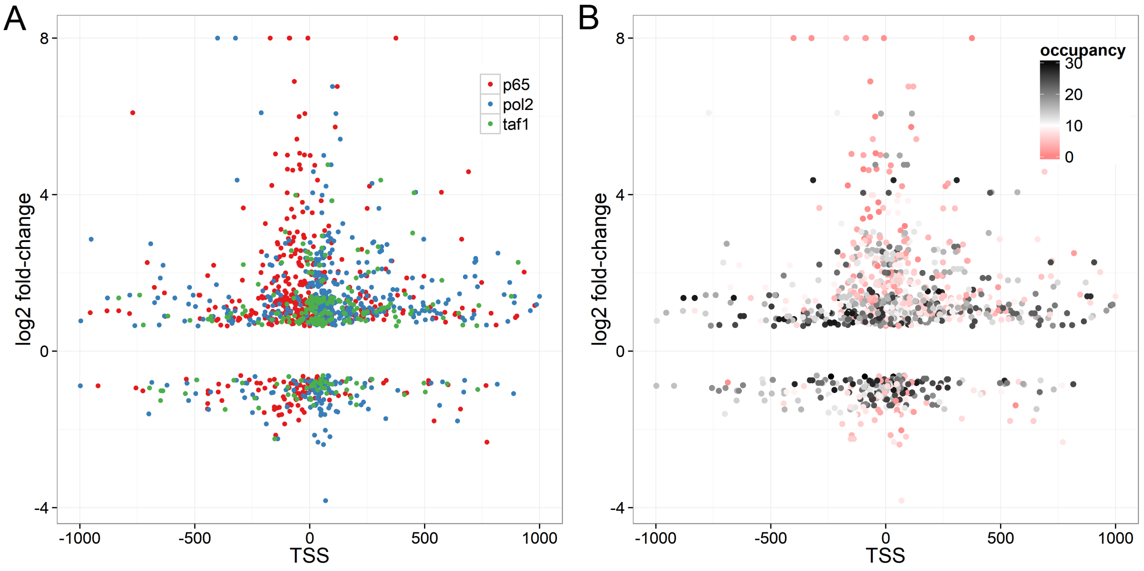

Summit positions of RNAPII (pol2), TAF1 (TAFII250), and p65 at the promoters of differentially expressed genes. Differential expression is shown on the Y-axis, summit position relative to the TSS is on the X-axis. (A) summits are color-coded by TF (B) summits are color-coded by the total occupancy of the promoter.

Figure S9: Promoters of stably expressed genes

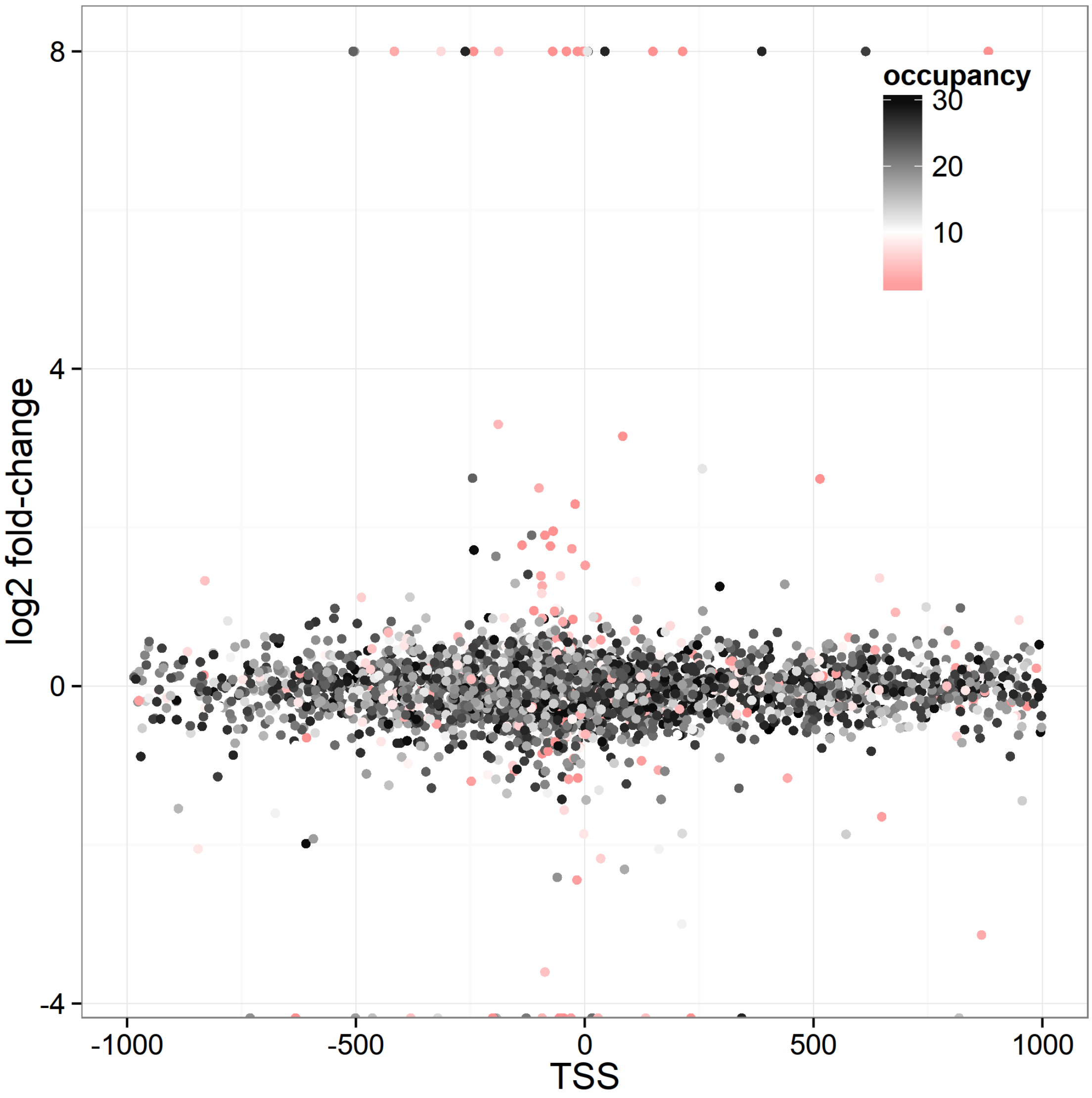

Summit positions of RNAPII (pol2), TAF1 (TAFII250), and p65 at the promoters of genes whose expression does not change in treatment. (complementary to Figure 6B). Differential expression is shown on the Y-axis, summit position relative to the TSS is on the X-axis. Summits are color-coded by the total occupancy of the promoter.

Figure S10: Importance of epigenetic marks in additive models of p65 binding

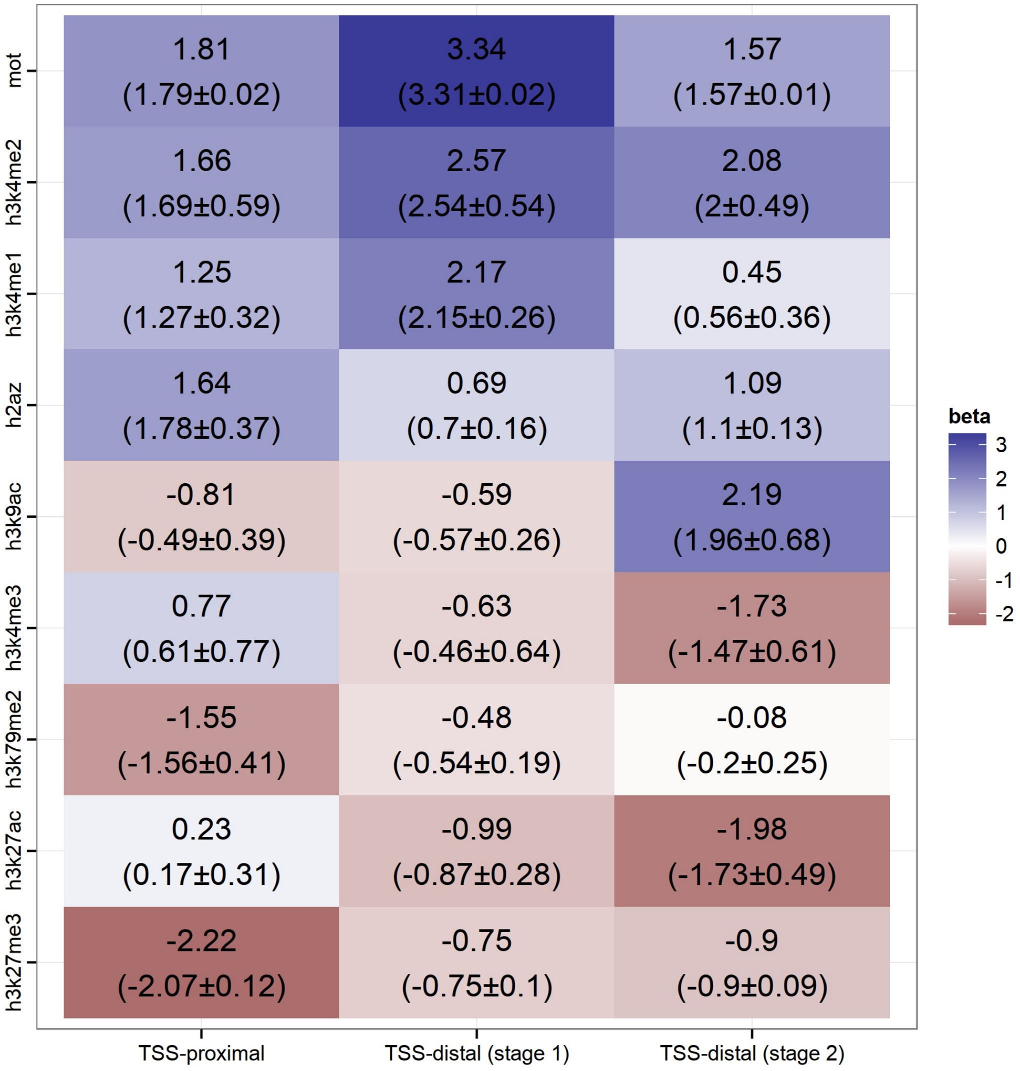

Multivariate additive models were trained separately on three classes of regulatory sites (TSS-proximal, TSS-distal *stage 1*, TSS-distal *stage 2*. Heatmap of standardized regression slopes for the 8 epigenetic marks together with the slope for NFkB motif presence (mot) are shown. Mean and standard deviation of slopes obtained from dropping each of the other epigenetic covariates from the model is indicated in brackets, and is a measure of the robustness of the estimate to model specification.

REFERENCES

1. Schaaf CA, Kwak H, Koenig A, Misulovin Z, Gohara DW, Watson A, Zhou Y, Lis JT, Dorsett D: **Genome-Wide Control of RNA Polymerase II Activity by Cohesin**. *PLoS Genet* 2013, **9**:e1003382.

2. Liu W-L, Coleman RA, Ma E, Grob P, Yang JL, Zhang Y, Dailey G, Nogales E, Tjian R: **Structures of three distinct activator-TFIID complexes**. *Genes Dev* 2009, **23**:1510–1521.

3. Smale ST: **Selective Transcription in Response to an Inflammatory Stimulus**. *Cell* 2010, **140**:833–844.

4. Agrawal R, Imieliński T, Swami A: **Mining association rules between sets of items in large databases**. *SIGMOD Rec* 1993, **22**:207–216.

5. Fournier-Viger P, Tseng VS: **Mining top-K non-redundant association rules**. In *Found Intell Syst*. Springer; 2012:31–40.

6. Anders S, Huber W: **Differential expression analysis for sequence count data**. *Genome Biol* 2010, **11**:R106.

7. Brunet J-P, Tamayo P, Golub TR, Mesirov JP: **Metagenes and molecular pattern discovery using matrix factorization**. *Proc Natl Acad Sci* 2004, **101**:4164–4169.

8. Lin C-J: **Projected Gradient Methods for Nonnegative Matrix Factorization**. *Neural Comput* 2007, **19**:2756–2779.
